# Impact of Lifestyle Modifications on Cancer Mortality: A Systematic Review and Meta-Analysis

**DOI:** 10.3390/medicina61020307

**Published:** 2025-02-10

**Authors:** Syed Arman Rabbani, Mohamed Anas Patni, Mohamed El-Tanani, Imran Rashid Rangraze, Adil Farooq Wali, Rasha Babiker, Shakta Mani Satyam, Yahia El-Tanani, Abdelrahman Adel Mohamed Shehata Almetwally

**Affiliations:** 1 RAK College of Pharmacy, RAK Medical and Health Sciences University, Ras Al Khaimah 11172, United Arab Emirates; 2 Translational and Medical Research Centre, RAK Medical and Health Sciences University, Ras Al Khaimah 11172, United Arab Emirates; 3 RAK College of Medical Sciences, RAK Medical and Health Sciences University, Ras Al Khaimah 11172, United Arab Emirates; 4 Royal Cornwall Hospital Trust, NHS, Truro TR1 3LJ, UK; 5 Faculty of Medicine, Cairo University, Giza 12613, Egypt

**Keywords:** cancer mortality, lifestyle modifications, dietary patterns, physical activity, smoking cessation, alcohol intake

## Abstract

*Background and Objectives*: Cancer survival poses significant challenges in oncology, with lifestyle modifications increasingly recognized as crucial in modifying patient outcomes post-diagnosis. This meta-analysis aims to systematically evaluate the impact of various lifestyle interventions on cancer survival across different types of cancer. *Methods*: A comprehensive literature search of electronic databases including PubMed, Scopus and Cochrane was performed to identify relevant studies up to 30 November 2024. Relevant studies were chosen and data were extracted and analyzed using SPSS Version 29.0 software. *Results*: Our systematic review included data from 98 studies involving a total of 1,461,834 cancer patients to evaluate the impact of lifestyle factors on cancer survival. Out of these, 64 studies were included in the meta-analysis. Our meta-analysis demonstrates that adherence to specific dietary patterns significantly improves cancer-specific outcomes. The Healthy Eating Index (HEI) diet was associated with a reduction in cancer-specific mortality (pooled log HR: −0.22; 95% CI: [−0.32, −0.12]; *p* < 0.001). Similar benefits were observed with the Mediterranean diet (aMED), which also reduced cancer mortality and recurrence (pooled log HR: −0.24; 95% CI: [−0.40, −0.07]; *p* < 0.001), and the Dietary Approaches to Stop Hypertension (DASH) diet (pooled log HR: −0.22; 95% CI: [−0.33, −0.12]; *p* < 0.001). Additionally, general dietary improvements were beneficial for breast cancer-specific mortality across 17 cohort studies (pooled log HR: −0.15; 95% CI: [−0.25, −0.06]; *p* < 0.001). Engaging in any form of physical activity post-diagnosis was associated with significant improvements in cancer-specific mortality or recurrence (pooled log HR: −0.31; 95% CI: [−0.38, −0.25]; *p* < 0.001). Participants who ceased smoking after diagnosis exhibited more favorable cancer outcomes (pooled log HR: −0.33; 95% CI: [−0.42, −0.24]; *p* < 0.001), with smoking cessation notably reducing cancer-specific mortality among lung cancer survivors (pooled log HR: −0.34; 95% CI: [−0.48, −0.20]; *p* < 0.001). Additionally, reducing alcohol intake post-diagnosis significantly improved cancer outcomes (pooled log HR: −0.26; 95% CI: [−0.33, −0.19]; *p* < 0.001). Alcohol moderation in gastrointestinal tract cancer survivors specifically decreased both cancer-specific mortality and recurrence (pooled log HR: −0.22; 95% CI: [−0.29, −0.15]; *p* < 0.001). *Conclusions*: Lifestyle modifications after cancer diagnosis significantly improve cancer-specific outcomes. Specific dietary patterns, increased physical activity, smoking cessation, and reduced alcohol intake are all associated with lower cancer-specific mortality. Integrating these lifestyle changes into oncology care may enhance patient survival and quality of life.

## 1. Introduction

Cancer remains one of the most formidable public health challenges globally, with its incidence consistently rising and becoming a major cause of morbidity and mortality worldwide. The World Health Organization (WHO) estimates that cancer is the second leading cause of death globally, contributing to approximately 10 million deaths in 2022 alone [1,2,3]. Cancer survival is a critical aspect of the disease’s trajectory, profoundly impacting patient prognosis and quality of life. Survival outcomes are influenced by a variety of factors, including the cancer type, stage at diagnosis, and the efficacy of initial treatment, with some cancers having more favorable survival rates than others [4,5].

Survival outcomes can be further categorized based on whether the cancer remains localized or spreads to other parts of the body. When cancer metastasizes, or spreads to distant sites, it often requires more intensive treatment and presents a greater challenge for long-term survival [4,5,6]. Amidst these challenges, lifestyle factors have increasingly been recognized as critical elements that can influence cancer survival. Research has consistently shown that certain lifestyle changes, implemented after a cancer diagnosis, can significantly impact overall survival and the quality of life. These factors include dietary habits, physical activity levels, smoking status, and alcohol consumption, each playing a distinct role in the patient’s health trajectory post-cancer treatment [7].

Dietary patterns and food choices play a crucial role in modulating cancer risk and recurrence. Diets rich in fruits, vegetables, whole grains, and lean proteins have been associated with a reduced risk of several types of cancer and improved survival rates [8]. Physical activity is another powerful lifestyle factor with a well-documented impact on cancer prognosis. Regular exercise helps reduce body fat, regulate hormone levels, and enhance immune function, all of which can contribute to reduced cancer recurrence rates and better survival [9,10,11].

Smoking is a well-established risk factor for many types of cancer, and continuing to smoke after a diagnosis can significantly worsen a patient’s prognosis. Smoking cessation is, therefore, a critical recommendation for cancer survivors. Studies have shown that quitting smoking at the time of diagnosis can dramatically improve survival rates and reduce the risk of recurrence [12]. Alcohol consumption is another modifiable risk factor that can influence cancer outcomes. Reducing alcohol intake post-diagnosis is advised to enhance health outcomes and reduce the risk of recurrence [13].

Despite the growing body of evidence supporting the impact of lifestyle modifications on cancer outcomes, substantial variability and contradictions exist across studies [14]. This inconsistency poses a challenge for oncologists and other healthcare providers aiming to offer evidence-based lifestyle recommendations to cancer survivors.

To address this, we conducted this systematic review and meta-analysis to evaluate and synthesize the existing research on the impact of different lifestyle modifications on cancer survival. This review analyzes the effects of dietary modifications, physical activity, smoking cessation, and alcohol consumption on cancer survival outcomes.

## 2. Methods

Our systematic review and meta-analysis was conducted according to the Preferred Reporting Items for Systematic Reviews and Meta-Analyses (PRISMA) guidelines. The review is registered with the OSF (https://doi.org/10.17605/OSF.IO/264SC, accessed on 5 February 2025).

### 2.1. Inclusion and Exclusion Criteria

Studies were selected based on predefined inclusion and exclusion criteria.

#### 2.1.1. Inclusion Criteria

We included in this analysis studies that were cohort studies or case-control studies reporting outcomes related to cancer mortality and that assessed one or more types of lifestyle modifications, such as dietary changes, physical activity, smoking cessation, or alcohol moderation, on the adult population with all cancer types.

#### 2.1.2. Exclusion Criteria

We excluded in the analysis studies that lacked a clear definition of cancer mortality or did not assess long-term survival. We also excluded editorials, letters, comments, or conference abstracts without full data, as well as studies with incomplete data or those that did not report effect sizes and confidence intervals. Additionally, studies focusing on pediatric populations were not included. Studies in languages other than English were excluded.

### 2.2. Search Strategy

To ensure a comprehensive and systematic collection of data relevant to the effects of lifestyle modifications on cancer mortality, an extensive literature search was conducted across multiple electronic databases. These databases included PubMed, Scopus, and the Cochrane Library. The search was designed to identify all relevant studies published from the inception of each database up to 30 November 2024, which was the cutoff date for study inclusion. This timeframe was selected to capture the most recent evidence while ensuring a thorough review of the available literature.

The search strategy incorporated a combination of MeSH terms and free-text terms to maximize the coverage. The key terms used were “cancer mortality”, “cancer survival”, “long-term survival”, “lifestyle modification”, “dietary intervention”, “physical activity”, “exercise”, “smoking cessation”, and “alcohol consumption”. These terms were used in various combinations with Boolean operators to ensure a comprehensive search. The search was supplemented by hand-searching the reference lists of included studies and relevant reviews to identify additional studies that might have been missed in the initial electronic search.

### 2.3. Selection Process

**Figure 1:**
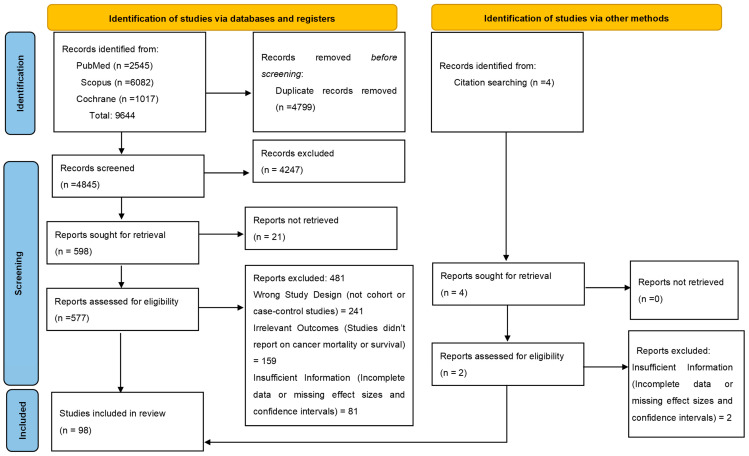
PRISMA flow diagram for the selection of studies.

The study selection was performed in two phases. After removing the duplicates, the titles and abstracts were screened by two independent reviewers as per the inclusion and exclusion criteria. Following this, full-text articles were assessed for eligibility. Reasons for exclusion were documented. Discrepancies were resolved through discussion or through consultation with the third reviewer. The selection process was documented in a PRISMA flow diagram, detailing the number of studies identified, screened, excluded, and included at each stage (Figure 1).

### 2.4. Data Extraction

Data from the selected studies were extracted independently by two reviewers using a standardized data extraction form to minimize bias. Discrepancies were resolved through discussion or consultation with a third reviewer when necessary. The extracted data included were study characteristics such as author(s), year of publication, country, and study design. Participant demographics were recorded, including age, sex, cancer type, and stage at diagnosis. We also documented the details of the lifestyle intervention, specifying the type, duration, and intensity of the intervention. The outcomes measured were cancer mortality and survival. Extracted data were entered into an Excel sheet.

### 2.5. Outcomes

The outcome measures selected for the analysis were cancer-specific mortality and cancer recurrence.

### 2.6. Quality Assessment 

Risk of bias assessment was undertaken independently by two reviewers. Discrepancies were discussed and resolved by consensus or through consultation with other reviewers. The quality and potential bias in individual studies were evaluated using Cochrane Risk of bias in Non-randomized Studies of Interventions. This tool is adaptable for observational studies and evaluates the risks across the following seven domains: confounding, participant selection, classification of interventions (or exposures), deviations from intended interventions (or exposures), missing data, measurement of outcomes, and selection of reported results.

### 2.7. Statistical Analysis

For this meta-analysis, the statistical analysis was performed with IBM SPSS version 29.0 for the 64 studies. Random-effects models were used throughout the analysis as a strategy to account for the expected heterogeneity across the studies that were included in the meta-analysis. This was aimed at providing more pointed and conservative estimations, knowing that the true effect sizes would not be the same, since the populations, interventions, and designs varied. The primary effect measure was the hazard ratio (HR) for disease-specific mortality as developed by cancer, and the level of confidence intervals (CIs) used to measure the level of accuracy was set at 95%. Studies accepted for the pooled analysis had log-transformed HRs, and their standard errors for the effect point estimates were extracted or calculated from the studies included. Heterogeneity was investigated with the I^2^ statistic, where values of 25%, 50%, and 75% were considered as representing low, moderate, and high heterogeneity between the studies, respectively. To evaluate the publication bias, the funnel plot asymmetries were visually examined. A *p*-value of <0.05 was interpreted as statistically significant in all of the tests. All of the analyses were two-tailed, as lifestyle modification interventions are assumed to work in both directions, affecting either side of the continuum.

This comprehensive methodology ensured that the conclusions drawn from this meta-analysis were robust, reliable, and informative, providing clear guidance for clinical practice concerning the impact of lifestyle modifications on cancer recurrence and survival.

## 3. Results

### 3.1. Study Selection

Initially, a comprehensive search across multiple databases yielded a total of 9644 potential studies. Upon closer inspection and the removal of duplicates, we screened 4845 studies by analyzing the titles and abstracts. This screening led to the exclusion of 4247 studies. We assessed 577 full-text articles for eligibility, of which 481 were further excluded due to reasons such as the wrong study design, irrelevant outcomes, and insufficient information. Ultimately, 98 studies met all of the inclusion criteria and were included in the review. The selection process followed the PRISMA (Preferred Reporting Items for Systematic Reviews and Meta-Analyses) guidelines, and a flowchart summarizing the study selection process is presented in Figure 1.

### 3.2. Study Characteristics

The final cohort of 98 studies included a diverse array of research designs, including 94 cohort studies and 4 case-control studies. These studies spanned from 2005 to 2024 and involved a broad geographical distribution, including 48 studies from North America, 25 from Europe, 14 from Asia, and 3 from Australia. Collectively, these studies encompassed a total participant count of 1,461,834, ranging from small-scale studies with 103 participants to large-scale studies involving up to 303,428 participants. The participants were adult cancer survivors. The types of cancer most frequently studied included breast cancer (23 studies), colorectal cancer (23 studies), all cancers (13 studies), lung cancer (9 studies), and prostate cancer (7 studies), among others. The characteristics of the studies included are summarized in Supplementary Tables S1–S4.

### 3.3. Qualitative Synthesis

The lifestyle interventions analyzed were categorized into the following four main types:

#### 3.3.1. Dietary Changes

Dietary changes were investigated in 43 [15,16,17,18,19,20,21,22,23,24,25,26,27,28,29,30,31,32,33,34,35,36,37,38,39,40,41,42,43,44,45,46,47,48,49,50,51,52,53,54,55,56,57] studies, focusing on interventions such as the increased intake of fruits and vegetables, reduced fat consumption, and adherence to specific dietary patterns like the Mediterranean diet. Among the 43 included studies, 10 studies [15,16,17,18,19,20,21,22,23,24] utilized the Healthy Eating Index (HEI) as a measure of high food quality. Only three studies [16,20,22] found an inverse relationship between the HEI and cancer-specific mortality. The other seven studies reported no association between the HEI and cancer-specific mortality, overall mortality, or recurrence.

Nine studies [28,29,30,31,34,35,36,37,38] employed the Prudent Diet Score as an index for high food quality. Lee et al. [30] identified an inverse relationship between the Prudent Diet Score and cancer-specific mortality. Guinter et al. [29] found that improvements in the DASH and Prudent Diet Scores from pre- to post-diagnosis were inversely associated with colorectal cancer (CRC)-specific mortality. Crowder et al. [31] suggested that adherence to a prudent diet before treatment may reduce the risk of chronic nutrition impact symptoms.

High-quality dietary indices, including the Alternative Healthy Eating Index (AHEI), Alternate Mediterranean Diet (aMED), and Dietary Approaches to Stop Hypertension (DASH), demonstrated mixed but promising associations. Eight studies [15,22,26,28,30,32,33,34] used the AHEI as an index for high food quality. Six of these studies reported an inverse relationship between the AHEI and cancer-specific mortality, while the remaining two [28,32] did not find a significant correlation. Eight studies [15,16,22,26,27,28,30,34] assessed the aMED as an index for high food quality. Two studies [15,30] found a significant inverse relationship between the aMED and cancer-specific mortality.

Eight studies [15,16,17,22,26,29,30,32] utilized the DASH as an index for high food quality. Four studies [15,17,29,30] reported an inverse relationship between the DASH and cancer-specific mortality. Ten studies [25,26,29,30,31,34,36,37,38,39] used the Western Diet Score as an index for low food quality. All of the studies indicated that the adherence to a Western diet was associated with adverse effects on cancer mortality and survival. Crowder et al. [31] found no association between the Western diet and nutrition impact symptoms.

Two studies [40,41] reported on maintaining a plant-based diet for cancer patients using the overall plant-based diet index (PDI), a healthful plant-based diet index (hPDI), and an unhealthful plant-based diet index (uPDI). Anyene et al. [40] reported that a healthful plant-based dietary pattern and unhealthful plant-based dietary pattern may reduce and increase the risk of non-breast-cancer mortality, respectively, whereas Ratjen et al. [41] revealed that the overall plant-based diet index displayed a significant, inverse association with all-cause mortality in colorectal cancer patients. However, Ollberding et al. [42] reported that maintaining a diet rich in fruits, vegetables, and starch after diagnosis of non-Hodgkin lymphoma (NHL) did not have an effect on overall survival.

Four studies reported on the World Cancer Research Fund/American Institute for Cancer Research (WCRF/AICR) recommendations in colorectal cancer patients [43,44,45,46]. In Van Zutphen et al. [43] and Romaguera et al. [45], a healthy lifestyle after a CRC diagnosis was associated with a decreased all-cause mortality risk and improved survival among CRC patients, respectively. However, in Song et al. [44], the post-diagnostic WCRF/AICR diet score was not statistically significantly associated with either colorectal cancer-specific or overall mortality. Two studies [47,48] studied the effect of adherence to the Mediterranean diet by using the Mediterranean diet score (MDS) and modified Mediterranean diet score (MMDS) [48] on cancer patients. They all found that maintaining a Mediterranean diet after the diagnosis of prostate, breast, and colorectal cancers, respectively, had a statistically significant association with better long-term overall survival.

Four studies [49,50,51,52] investigated the effect of an anti-inflammatory diet using the dietary inflammatory index (DII) [51,52] and energy-adjusted dietary inflammatory index (E-DII) [49,50] on colorectal cancer (CRC), and breast and prostate cancer (PCa) patients. The studies found that the dietary pattern with the most anti-inflammatory potential was associated with a decrease in all-cause mortality among postmenopausal women after diagnosis with CRC [49], a lower risk of cardiovascular mortality [50], and a decrease in the risk of cancer recurrence and overall mortality in patients with breast cancer [51]. However, Zucchetto et al. [52] reported that DII scores were not significantly associated with the all-cause mortality of PCa patients. Three studies [53,54,55] reported on the effect of a hyperinsulinemic state on cancer survival using the following different indices: the dietary insulin index [55], empirical dietary index for hyperinsulinemia (EDIH) [56], and diabetes risk reduction diet (DRRD) [57]. In colorectal cancer patients, higher dietary insulin scores after colorectal cancer diagnosis were associated with a statistically significant increase in colorectal cancer-specific and overall mortality, indicating poorer survival [53,55], and reduced mortality after breast cancer diagnosis [54].

The relation between diets consistent with the American Cancer Society (ACS) recommendations and cancer outcomes was assessed by McCullough et al. [56], revealing that a diet consistent with ACS recommendations, either pre-diagnosis or post-diagnosis, did not have a statistically significant effect on cancer-specific mortality in patients with breast cancer. Ferrohna et al. [57] evaluated pre-diagnostic dietary patterns in gastric cancer patients, categorizing the intake into the following three groups: Pattern I (high dairy, fruits, and salads/vegetables; low meat/alcohol), Pattern II (low consumption of dairy, fish, produce, and meat), and Pattern III (high in most food groups; minimal vegetable soup). Pattern III demonstrated a statistically significant association with improved prognosis in cases classified as regional spread.

#### 3.3.2. Physical Activity

Physical activity was investigated in 20 studies [10,14,34,58,59,60,61,62,63,64,65,66,67,68,69,70,71,72,73,74], focusing on the relationship between physical activity and cancer survival outcomes across various cancer types. The studies varied in design, participant demographics, and follow-up durations, yielding valuable insights into how physical activity influences survival rates among cancer patients. Among the included studies, seven studies [34,58,62,67,68,73,74] specifically highlighted the beneficial effects of adhering to physical activity guidelines post-diagnosis. These studies consistently demonstrated that engaging in regular physical activity is associated with improved survival rates in breast, colorectal, ovarian, and other cancer types.

Six studies [14,59,60,65,69,71] explored the association between varying intensities of physical activity and cancer survival. Hamer et al. [60] found that vigorous physical activity significantly reduced the cancer mortality risk compared to mild activity levels. Conversely, Ueshima et al. [14] reported unclear relationships, suggesting that the impact of physical activity may depend on specific cancer types or patient characteristics.

Seven studies [10,14,61,66,70,72,74] assessed the role of sedentary behavior in conjunction with physical activity levels. Cao et al. [70] identified that prolonged sitting combined with low physical activity significantly increased the risk of death from all causes, including cancer. This indicates that reducing sedentary time while increasing physical activity could be crucial for improving survival outcomes.

Five studies [34,63,64,67,73] highlighted the importance of the timing of physical activity in relation to diagnosis. For example, Van Blarigan et al. [34] emphasized that regular physical activity after a stage III colon cancer diagnosis was linked to improved survival, reinforcing the notion that post-diagnosis lifestyle changes can have a substantial impact on outcomes.

Overall, the synthesis of these studies indicates a robust association between physical activity and improved cancer survival, underscoring the need for guidelines promoting physical activity as a modifiable risk factor in cancer care. This is particularly relevant for patients across various cancer types, including breast, colorectal, and ovarian cancers, emphasizing the importance of integrating physical activity into treatment and survivorship plans.

#### 3.3.3. Smoking Cessation

Smoking cessation was evaluated in 19 studies focusing on its impact on survival outcomes in cancer patients [71,74,75,76,77,78,79,80,81,82,83,84,85,86,87,88,89,90,91,92]. Among the included studies, six studies [71,75,76,77,78,83] highlighted that patients who quit smoking after a cancer diagnosis experienced significantly improved overall survival compared to those who continued smoking. Five studies [77,79,80,82,85,87] reported that ongoing smoking at the time of diagnosis was linked to poorer survival outcomes across various cancer types, including lung and colorectal cancers. Koshiaris et al. [77] indicated that individuals with lung cancer who quit smoking had a lower risk of mortality compared to those who continued smoking.

Five studies [79,84,86,88,91] examined the effects of smoking cessation on specific cancer types, noting that quitting smoking was associated with reduced cancer-related mortality. Four studies [81,89,90,92] investigated the timing of smoking cessation, with findings suggesting that quitting before diagnosis or early in the treatment process significantly correlated with better survival rates. Linhas et al. [90] found that smoking cessation before chemotherapy was associated with improved overall survival in non-small-cell lung cancer patients.

In summary, the evidence strongly supports that smoking cessation is associated with improved survival outcomes in cancer patients, underscoring the importance of quitting smoking at any stage of the disease.

#### 3.3.4. Alcohol Moderation

Alcohol moderation was investigated in 20 studies focusing on the relationship between alcohol consumption and various health outcomes [24,71,93,94,95,96,97,98,99,100,101,102,103,104,105,106,107,108,109,110,111], particularly cancer-specific mortality and overall mortality. Among the studies, nine studies [24,71,93,94,97,101,102,103,104] identified that light alcohol consumption was associated with a decreased risk of certain cancers, notably breast cancer. Eight studies [94,98,99,100,101,105,106,107] reported that heavy alcohol consumption elevates the risk of overall mortality and specific cancers, such as liver and colorectal cancers. Koyama et al. [101] highlighted that heavy drinkers consuming more than 46 g/day of ethanol faced an increased risk of death compared to non-drinkers.

Six studies [95,102,104,108,110,111] examined post-diagnosis alcohol consumption and its association with increased mortality rates, particularly for prostate cancer. Farris et al. [98] noted that post-diagnosis consumption was linked to higher mortality rates in prostate cancer patients. Five studies [96,98,106,108,109] explored the moderation of alcohol consumption as a protective factor against cancer-related mortality. Jankhotkaew et al. [108] suggested that moderate alcohol intake may mitigate the risks associated with specific cancers, emphasizing the need for moderation in drinking behaviors.

Pre-diagnosis alcohol consumption has a non-linear association with increased breast cancer-specific mortality and may elevate the cancer-specific mortality risk in colorectal cancers. Light alcohol consumption significantly lowers the risk of all-cause mortality. However, heavy drinking before diagnosis is linked to poorer survival rates after a colorectal cancer diagnosis compared to light drinking. The protective benefits of light alcohol consumption might be limited to wine, and could vary based on age and the presence of diabetes mellitus. In contrast, post-diagnosis alcohol consumption is associated with increased mortality, particularly for prostate cancer. Heavy drinkers consuming more than 46 g/day of ethanol have a higher risk of death from oropharyngeal cancers compared to non-drinkers, regardless of gender.

Overall, the evidence suggests a complex relationship between alcohol consumption and health outcomes, with light consumption potentially offering some protective benefits, while heavy consumption remains a significant risk factor for various cancers and overall mortality.

### 3.4. Quantitative Synthesis

The meta-analysis utilized both fixed-effects and random-effects models to account for variability across studies. The analysis revealed significant associations between lifestyle modifications and cancer outcomes:

#### 3.4.1. Dietary Changes

**Figure 2:**
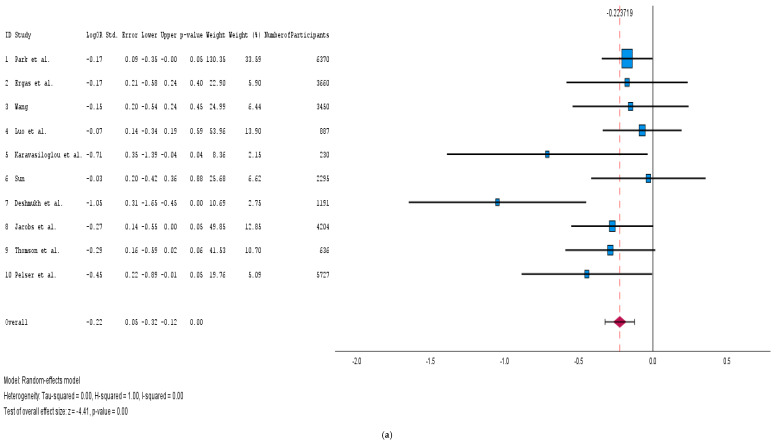

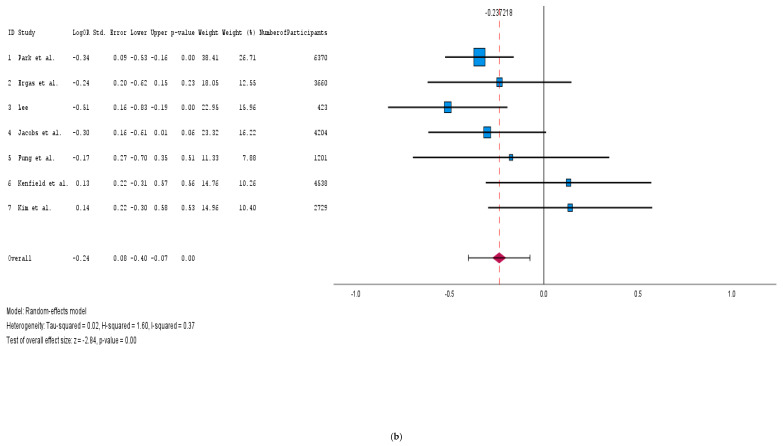

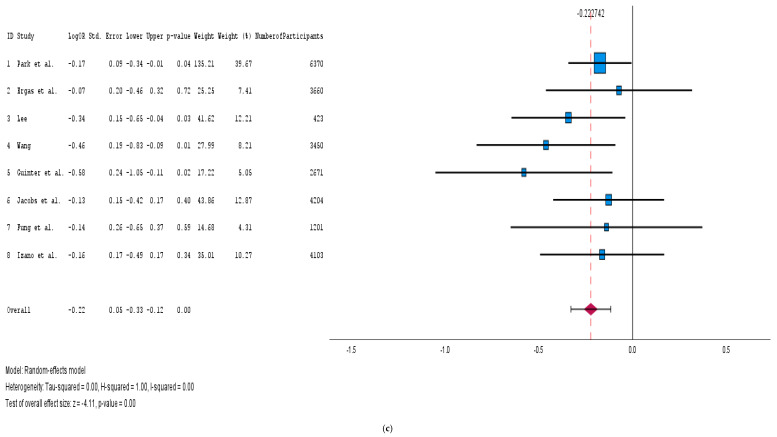

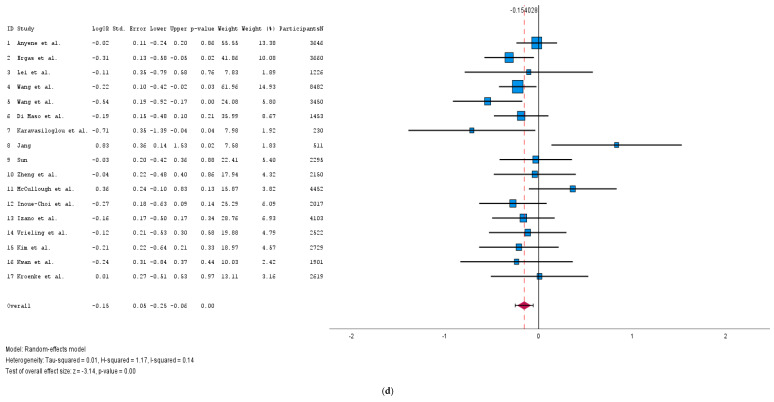
(**a**): Forest plots of the HEI diet impact on cancer survival [15,16,17,18,19,20,21,22,23,24]. (**b**): Forest plots of the aMED diet impact on cancer survival [15,16,22,26,27,28,30]. (**c**): Forest plots of the DASH diet impact on cancer survival [15,16,17,22,29,30,32]. (**d**): Forest plots of the diet (any) impact on breast cancer survival outcomes [16,17,19,20,28,32,37,38,39,40,46,47,49,51,56].

We conducted an analysis of 10 cohort studies, encompassing a total of 28,650 cancer patients, to investigate the effects of adherence to the Healthy Eating Index (HEI) diet on cancer-specific mortality. The forest plot, which we generated using a random-effects model (Figure 2a), indicated that the adherence to the HEI diet post-cancer diagnosis was associated with significantly improved outcomes in terms of cancer-specific mortality (pooled log HR: −0.22; 95% CI: [−0.32, −0.12]; *p* < 0.001). The heterogeneity among the studies was very low (I^2^ = 0%), suggesting a low variability among the studies. Furthermore, the funnel plot (Supplementary Figure S1) demonstrated no signs of publication bias across the studies, as the plot was asymmetric.

We analyzed seven cohort studies, encompassing 23,125 cancer patients, to assess the impact of adherence to the aMED diet on cancer-specific mortality. The forest plot, which we generated using a random-effects model (Figure 2b), demonstrates that following the aMED diet after a cancer diagnosis is associated with better outcomes in terms of cancer-specific mortality and recurrence (pooled log HR: −0.24; 95% CI: [−0.40, −0.07]; *p* < 0.001). The random-effects model showed no heterogeneity (I^2^ = 0%), indicating a high consistency among the studies. Additionally, the funnel plot (Supplementary Figure S2) reveals no indications of publication bias across the studies.

We examined eight cohort studies, involving a total of 26,082 cancer patients, to evaluate the effects of adherence to the DASH diet on cancer-specific mortality. The forest plot, which we generated using a random-effects model (Figure 2c), indicates that following the DASH diet after a cancer diagnosis improves outcomes in both cancer-specific and overall mortality (pooled log HR: −0.22; 95% CI: [−0.33, −0.12]; *p* < 0.001). The random-effects model shows a very low heterogeneity (I^2^ = 0%), suggesting a high level of consistency across the studies. Moreover, the funnel plot (Supplementary Figure S3) shows no signs of publication bias.

We also generated forest and funnel plots after including 17 cohort studies conducted on the effect of any form of diet on cancer survival in breast cancer patients. The forest plot we created using a random-effects model (Figure 2d) demonstrated that any form of diet had a favorable outcome for breast cancer-specific mortality (pooled log HR: −0.15; 95% CI: [−0.25, −0.06]; *p* < 0.001). The heterogeneity was very low (I^2^ = 14%; *p* < 0.001), indicating consistency among the studies. Additionally, the funnel plot revealed symmetry, suggesting the absence of publication bias (Supplementary Figure S4).

#### 3.4.2. Physical Activity

**Figure 3:**
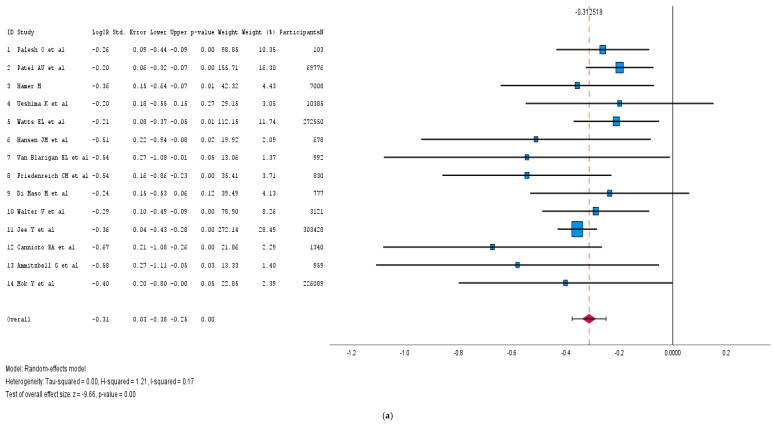

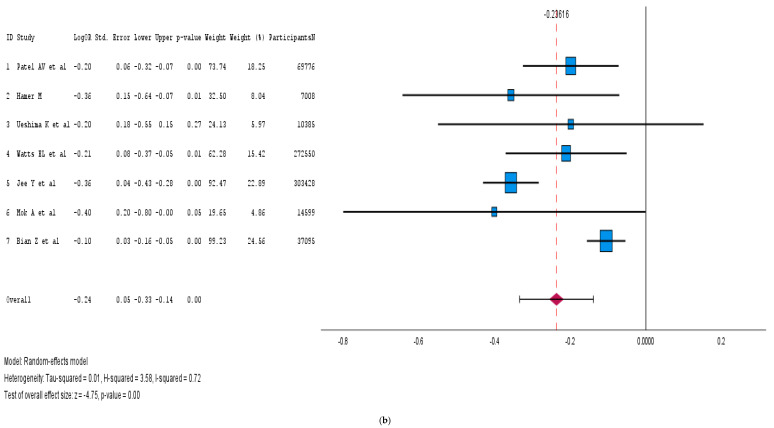
(**a**): Forest plot of the physical activity impact on cancer survival outcomes [14,34,58,59,60,61,62,63,64,65,66,67,68,69]. (**b**): Forest plot Physical activity with all cancer survival only [14,59,60,61,66,69,71].

We explored 14 cohort studies, encompassing a total of 898,036 cancer patients, to assess the impact of any form of physical activity on cancer-specific mortality. The forest plot we created using a random-effects model (Figure 3a) shows that any form of physical activity has a favorable outcome for cancer-specific mortality or recurrence (pooled log HR: −0.31; 95% CI: [−0.38, −0.25]; *p* < 0.001). The heterogeneity is very low (I^2^ = 0%), indicating consistency across the studies. There seemed to be the presence of publication bias among the different studies included, as shown in Supplementary Figure S5, which suggests that studies with favorable outcomes were published more often.

We also generated forest and funnel plots after including seven cohort studies conducted on the effect of physical activity on cancer-specific mortality for all cancer patients, excluding studies that measured the impact of physical activity on specific cancers. The forest plot we created using a random-effects model (Figure 3b) demonstrated that any form of physical activity had a favorable outcome for overall cancer-specific mortality or recurrence (pooled log HR: −0.24; 95% CI: [−0.33, −0.14]; *p* < 0.001). The heterogeneity was greater (I^2^ = 72%), indicating inconsistency among the studies, which may be because of studies which included different cancer types, stages, and treatment regimens, or variation in the types, intensity, and frequency of physical activity across the studies. Additionally, the funnel plot revealed no asymmetry, suggesting the absence of publication bias (Supplementary Figure S6).

#### 3.4.3. Smoking Cessation

**Figure 4:**
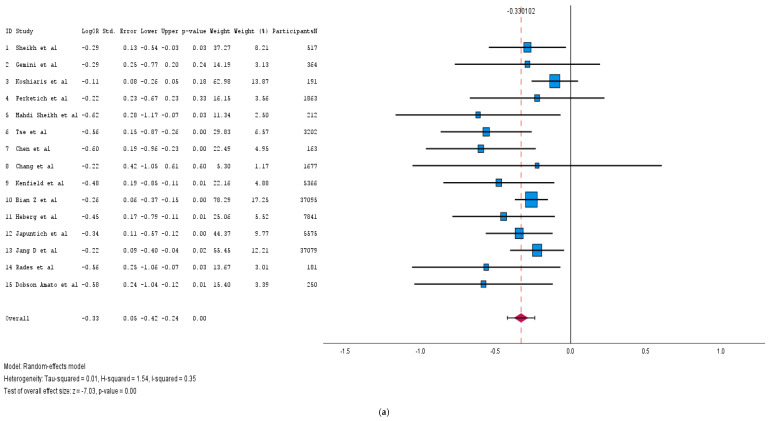

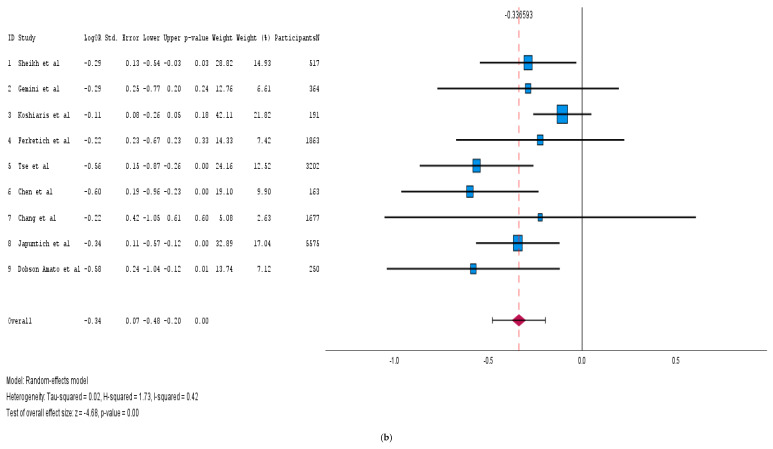
(**a**): Forest plot of the impact of smoking cessation on cancer survival outcomes [71,75,76,77,78,79,80,81,82,83,84,85,86,87,88]. (**b**): Forest plot for impact of smoking cessation with lung cancer survival only [75,76,77,78,80,81,82,85,88].

We identified 15 cohort studies, incorporating a total of 101,576 patients, to assess the impact of smoking cessation on cancer-specific mortality. We generated a forest plot (Figure 4a) including all of these studies, and observed that participants who quit smoking after diagnosis had significantly more favorable cancer outcomes (pooled log HR: −0.33; 95% CI: [−0.42, −0.24]; *p* < 0.001). The results across the studies were moderately consistent (I^2^ = 35%). The funnel plot (Supplementary Figure S7) revealed no publication bias among the studies.

We then focused on nine cohort studies assessing the effect of smoking cessation on cancer-specific mortality in lung cancer survivors, excluding studies focusing on other cancer types. The forest plot (Figure 4b), utilizing a random-effects model, demonstrated that smoking cessation significantly reduced cancer-specific mortality in lung cancer survivors (pooled log HR: −0.34; 95% CI: [−0.48, −0.20]; *p* < 0.001). While the analysis revealed moderate heterogeneity (I^2^ = 42%), indicating some variability among the studies, the funnel plot (Supplementary Figure S8) showed signs of symmetry, pointing to a low potential for publication bias.

#### 3.4.4. Alcohol Moderation

**Figure 5:**
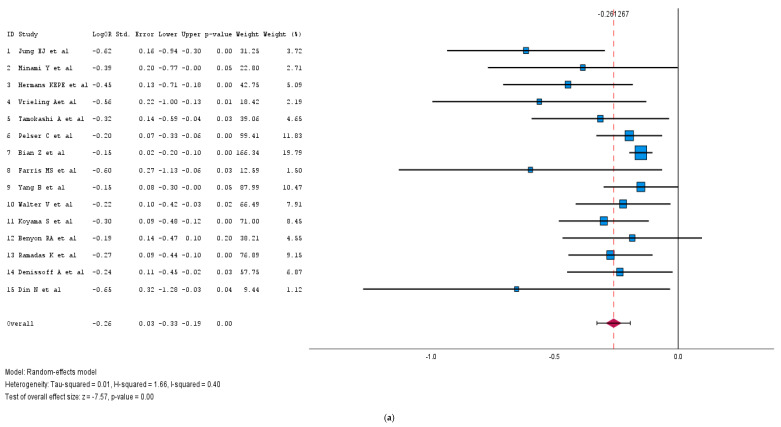

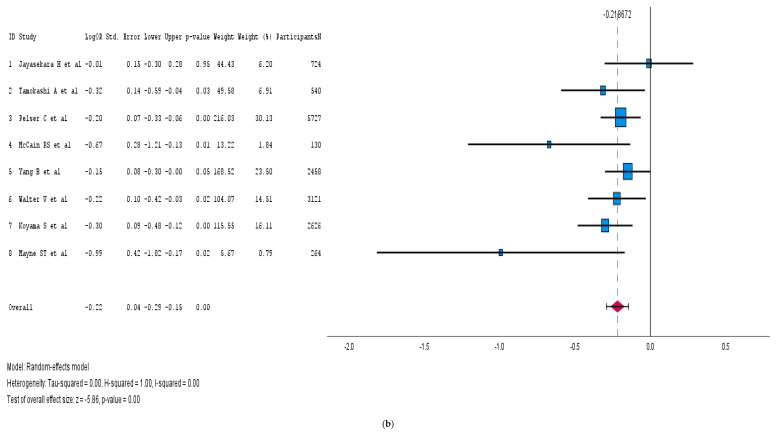
(**a**): Forest plot of the impact of the reduction in alcohol intake on cancer survival [24,71,93,94,95,96,97,98,99,100,101,102,103,104,105]. (**b**): Alcohol moderation on cancer-specific mortality among gastrointestinal tract cancer survivors [24,97,99,100,101,107,109,111].

We conducted a meta-analysis of 15 cohort studies, involving 365,218 patients, to evaluate the impact of alcohol moderation on cancer-specific mortality. A forest plot (Figure 5a) was constructed using data from these studies, revealing that individuals who reduced their alcohol intake post-diagnosis experience significantly improved their cancer outcomes (pooled log HR: −0.26; 95% CI: [−0.33, −0.19]; *p* < 0.001). The results demonstrated moderate heterogeneity (I^2^ = 40%). Additionally, a funnel plot (Supplementary Figure S9) indicated some evidence of publication bias.

Subsequently, we focused on eight cohort studies specifically examining the effects of alcohol moderation on cancer-specific mortality among gastrointestinal tract cancer survivors, excluding studies related to other cancer types. The forest plot (Figure 5b), generated using a random-effects model, showed that alcohol moderation significantly decreased both cancer-specific mortality and recurrence in this group (pooled log HR: −0.22; 95% CI: [−0.29, −0.15]; *p* < 0.001). The analysis indicated a very low heterogeneity (I^2^ = 0%), suggesting a high consistency among the studies. The funnel plot (Supplementary Figure S10) displayed symmetry, indicating a low likelihood of publication bias.

## 4. Discussion

This systematic review and meta-analysis on lifestyle interventions and their impact on cancer outcomes reveals significant insights derived from both qualitative and quantitative syntheses. In the qualitative synthesis, lifestyle interventions were categorized into dietary changes, physical activity, smoking cessation, and alcohol moderation, with each category demonstrating its potential effects on cancer-specific mortality and recurrence.

Numerous studies have independently examined the effects of alcohol moderation, smoking cessation, physical activity, and dietary patterns on cancer-specific mortality and recurrence. For instance, individual studies have consistently demonstrated that reducing alcohol intake post-diagnosis improves cancer outcomes, while smoking cessation has been linked to decreased cancer-specific mortality and recurrence, particularly in lung cancer survivors. Similarly, physical activity has been associated with favorable cancer outcomes, and adherence to healthy dietary patterns, such as the HEI, aMED, and DASH diets, has shown significant benefits in reducing cancer-specific mortality and recurrence. However, our study is unique in synthesizing these four lifestyle factors together, providing a comprehensive overview of their combined impact on cancer outcomes.

Dietary changes were examined across 46 studies, focusing on various dietary patterns, such as the Healthy Eating Index (HEI) and the Mediterranean diet. While only three studies found an inverse relationship between the HEI and cancer-specific mortality, nine studies using the Prudent Diet Score indicated a beneficial association with reduced mortality, particularly in colorectal cancer. Other dietary indices, including the Alternative Healthy Eating Index (AHEI) and the Dietary Approaches to Stop Hypertension (DASH), also showed promising results, reinforcing the importance of high food quality in cancer survival outcomes. Conversely, adherence to a Western diet was consistently linked to poorer outcomes, highlighting the negative impact of low-quality dietary patterns.

Physical activity was evaluated in 20 studies, with 10 studies specifically underscoring the benefits of meeting physical activity guidelines post-diagnosis. Regular physical activity was associated with improved survival rates across multiple cancer types, including breast and colorectal cancer. The analysis revealed that vigorous activity notably reduced the mortality risk compared to mild levels, while sedentary behavior correlated with increased mortality risk, emphasizing the necessity of reducing sedentary time to enhance cancer survival.

Smoking cessation was supported by evidence from 21 studies, which demonstrated that patients who quit smoking after their cancer diagnosis had significantly better survival outcomes. Timing was crucial; quitting before diagnosis or early in the treatment process was associated with improved survival rates, particularly for lung cancer patients.

Lastly, alcohol moderation was investigated in 20 studies, indicating a complex relationship between alcohol consumption and health outcomes. While light alcohol consumption was linked to decreased cancer risk, heavy drinking consistently elevated the mortality risk across several cancer types. Notably, post-diagnosis alcohol consumption correlated with increased mortality rates, particularly in prostate cancer, emphasizing the need for moderation following diagnosis.

In the quantitative synthesis, the meta-analysis results reinforced the findings from the qualitative analysis, revealing significant associations between lifestyle modifications and improved cancer outcomes. For instance, adherence to the HEI diet demonstrated a hazard ratio log HR to be −0.22 for cancer-specific mortality, indicating a decreased risk of mortality in patients adopting this diet. Similarly, the aMED and DASH diets showed log HRs of −0.24 and −0.22, respectively, supporting the role of these dietary patterns in enhancing survival rates.

Physical activity was shown to have a favorable effect on cancer-specific mortality (log HR: −0.31), while smoking cessation yielded a significant reduction in cancer-specific mortality and recurrence (log HR: −0.33). Alcohol moderation also revealed a positive impact, with a log HR of −0.26 for those reducing intake post-diagnosis.

Clinically, our study underscores the importance of a multifaceted approach to cancer survivorship care. By integrating alcohol moderation, smoking cessation, physical activity, and adherence to healthy dietary patterns, healthcare providers can offer more holistic and effective strategies to improve cancer-specific outcomes. Academically, this study fills a critical gap in the literature by providing a consolidated analysis of these lifestyle factors, which were previously studied in isolation. This comprehensive approach not only enhances our understanding of the individual and combined effects of these factors but also sets a foundation for future research to explore synergistic interventions.

One of the primary strengths of our study is the comprehensive nature of the systematic review, which includes a large sample size across multiple cohort studies, thereby increasing the generalizability of our findings. Additionally, the use of random-effects models and the assessment of publication bias through funnel plots add robustness to our analysis.

This comprehensive meta-analysis also has a number of limitations that must be recognized. There is potential publication bias, represented by the asymmetry of the funnel plots, which could mean the over-representation of positive-results studies, especially regarding physical activity, thus inflating the apparent effects of lifestyle interventions. Observational studies often could not be fully adjusted for confounding factors such as socioeconomic status, health behaviors at baseline, and comorbid conditions; thus, biases may arise. High heterogeneity, for instance, in physical activity, is up to I^2^ = 72%, reflecting variability in the study designs, populations, and measurement methods, which may affect the generalizability of the findings. Many studies relied on self-reported data for lifestyle behaviors; hence, the results cannot avoid recall and reporting biases. The variation in the timing of interventions, for example, since the diagnosis, was also irregularly analyzed, which can modify the observed associations. These factors together make the case for a cautious interpretation and stronger research in the future.

## 5. Conclusions

Our systematic review and meta-analysis highlights the significant benefits of alcohol moderation, smoking cessation, physical activity, and adherence to healthy dietary patterns on cancer-specific mortality. These findings advocate for a comprehensive lifestyle modification approach in cancer survivorship care, emphasizing the need for integrated interventions that address multiple lifestyle factors simultaneously.

Healthcare providers should encourage cancer patients to adopt a holistic lifestyle modification strategy that includes reducing alcohol intake, quitting smoking, engaging in regular physical activity, and following healthy dietary patterns. Such an approach has the potential to significantly improve cancer-specific outcomes and enhance overall survivorship quality.

## Data Availability

The data used to support the findings of this study are included within the article.

## Supplementary Materials

The following supporting information can be downloaded at: https://www.mdpi.com/article/10.3390/medicina61020307/s1, Table S1: Characteristics of studies associated with Dietary changes; Table S2: Characteristics of studies associated with Physical Activity; Table S3: Characteristics of studies associated with Smoking Cessation; Table S4: Characteristics of studies associated with Alcohol Moderation; Figure S1: Funnel plots to assess publication bias in studies assessing HEI diet impact on cancer survival; Figure S2: Funnel plots to assess publication bias in studies assessing aMED diet impact on cancer survival; Figure S3: Funnel plots to assess publication bias in studies assessing DASH diet impact on cancer survival; Figure S4: Funnel plots to assess publication bias in studies assessing diet impact on breast cancer survival; Figure S5: Funnel plot to assess publication bias in studies assessing physical activity impact on cancer outcomes; Figure S6: Funnel plot to assess publication bias in studies assessing physical activity impact on all cancer survival outcomes only; Figure S7: Funnel plot to assess publication bias in studies assessing smoking cessation impact on all cancer survival; Figure S8: Funnel plot to assess publication bias in studies assessing smoking cessation impact on lung cancer survival; Figure S9: Funnel plot to assess publication bias in studies assessing alcohol impact on all cancer survival; Figure S10: Funnel plot to assess publication bias in studies assessing alcohol impact on survival in gastrointestinal tract cancers.

## References

[1] Sung H., Ferlay J., Siegel R.L., Laversanne M., Soerjomataram I., Jemal A., Bray F. (2021). Global Cancer Statistics 2020: GLOBOCAN Estimates of Incidence and Mortality Worldwide for 36 Cancers in 185 Countries. CA Cancer J. Clin..

[2] Cancer. https://www.who.int/news-room/fact-sheets/detail/cancer.

[3] Zhao J., Xu L., Sun J., Song M., Wang L., Yuan S., Zhu Y., Wan Z., Larsson S., Tsilidis K. (2023). Global Trends in Incidence, Death, Burden and Risk Factors of Early-Onset Cancer from 1990 to 2019. BMJ Oncol..

[4] Fabian D., Guillermo Prieto Eibl M.D.P., Alnahhas I., Sebastian N., Giglio P., Puduvalli V., Gonzalez J., Palmer J.D. (2019). Treatment of Glioblastoma (GBM) with the Addition of Tumor-Treating Fields (TTF): A Review. Cancers.

[5] Corrado G., Salutari V., Palluzzi E., Distefano M.G., Scambia G., Ferrandina G. (2017). Optimizing Treatment in Recurrent Epithelial Ovarian Cancer. Expert. Rev. Anticancer. Ther..

[6] Cancer Treatment|Survivor Facts & Figures. https://www.cancer.org/research/cancer-facts-statistics/survivor-facts-figures.html.

[7] Vijayvergia N., Denlinger C.S. (2015). Lifestyle Factors in Cancer Survivorship: Where We Are and Where We Are Headed. J. Pers. Med..

[8] Jochems S.H.J., Van Osch F.H.M., Bryan R.T., Wesselius A., van Schooten F.J., Cheng K.K., Zeegers M.P. (2018). Impact of Dietary Patterns and the Main Food Groups on Mortality and Recurrence in Cancer Survivors: A Systematic Review of Current Epidemiological Literature. BMJ Open.

[9] Physical Activity and Cancer Fact Sheet-NCI. https://www.cancer.gov/about-cancer/causes-prevention/risk/obesity/physical-activity-fact-sheet.

[10] Friedenreich C.M., Stone C.R., Cheung W.Y., Hayes S.C. (2020). Physical Activity and Mortality in Cancer Survivors: A Systematic Review and Meta-Analysis. JNCI Cancer Spectr..

[11] Thomas R., Kenfield S.A., Yanagisawa Y., Newton R.U. (2021). Why Exercise Has a Crucial Role in Cancer Prevention, Risk Reduction and Improved Outcomes. Br. Med. Bull..

[12] Smoking Cessation and Cancer Survivorship|Tobacco and E-Cigarettes|JAMA|JAMA Network. https://jamanetwork.com/journals/jama/fullarticle/2771610.

[13] Alcohol and Cancer Risk Fact Sheet-NCI. https://www.cancer.gov/about-cancer/causes-prevention/risk/alcohol/alcohol-fact-sheet.

[14] Ueshima K., Ishikawa-Takata K., Yorifuji T., Suzuki E., Kashima S., Takao S., Sugiyama M., Ohta T., Doi H. (2010). Physical Activity and Mortality Risk in the Japanese Elderly: A Cohort Study. Am. J. Prev. Med..

[15] Park S.-Y., Kang M., Shvetsov Y.B., Setiawan V.W., Boushey C.J., Haiman C.A., Wilkens L.R., Le Marchand L. (2022). Diet Quality and All-Cause and Cancer-Specific Mortality in Cancer Survivors and Non-Cancer Individuals: The Multiethnic Cohort Study. Eur. J. Nutr..

[16] Ergas I.J., Cespedes Feliciano E.M., Bradshaw P.T., Roh J.M., Kwan M.L., Cadenhead J., Santiago-Torres M., Troeschel A.N., Laraia B., Madsen K. (2021). Diet Quality and Breast Cancer Recurrence and Survival: The Pathways Study. JNCI Cancer Spectr..

[17] Wang F., Cai H., Gu K., Shi L., Yu D., Zhang M., Zheng W., Zheng Y., Bao P., Shu X.-O. (2020). Adherence to Dietary Recommendations Among Long-Term Breast Cancer Survivors and Cancer Outcome Associations. Cancer Epidemiol. Biomark. Prev..

[18] Luo Y., Zhang Y.-J., Zhang D.-M., Yishake D., Liu Z.-Y., Chen M.-S., Wang F., Zhou Z.-G., Long J.-A., Zhong R.-H. (2020). Association between Dietary Patterns and Prognosis of Hepatocellular Carcinoma in the Guangdong Liver Cancer Cohort Study. Hepatol. Res..

[19] Karavasiloglou N., Pestoni G., Faeh D., Rohrmann S. (2019). Post-Diagnostic Diet Quality and Mortality in Females with Self-Reported History of Breast or Gynecological Cancers: Results from the Third National Health and Nutrition Examination Survey (NHANES III). Nutrients.

[20] Sun Y., Bao W., Liu B., Caan B.J., Lane D.S., Millen A.E., Simon M.S., Thomson C.A., Tinker L.F., Van Horn L.V. (2018). Changes in Overall Diet Quality in Relation to Survival in Postmenopausal Women with Breast Cancer: Results from the Women’s Health Initiative. J. Acad. Nutr. Diet..

[21] Deshmukh A.A., Shirvani S.M., Likhacheva A., Chhatwal J., Chiao E.Y., Sonawane K. (2018). The Association Between Dietary Quality and Overall and Cancer-Specific Mortality Among Cancer Survivors, NHANES III. JNCI Cancer Spectr..

[22] Jacobs S., Harmon B.E., Ollberding N.J., Wilkens L.R., Monroe K.R., Kolonel L.N., Le Marchand L., Boushey C.J., Maskarinec G. (2016). Among 4 Diet Quality Indexes, Only the Alternate Mediterranean Diet Score Is Associated with Better Colorectal Cancer Survival and Only in African American Women in the Multiethnic Cohort. J. Nutr..

[23] Thomson C.A., E Crane T., Wertheim B.C., Neuhouser M.L., Li W., Snetselaar L.G., Basen-Engquist K.M., Zhou Y., Irwin M.L. (2014). Diet Quality and Survival after Ovarian Cancer: Results from the Women’s Health Initiative. J. Natl. Cancer Inst..

[24] Pelser C., Arem H., Pfeiffer R.M., Elena J.W., Alfano C.M., Hollenbeck A.R., Park Y. (2014). Pre-Diagnostic Lifestyle Factors and Survival after Colon and Rectal Cancer Diagnosis in the NIH-AARP Diet and Health Study. Cancer.

[25] Arthur A.E., Peterson K.E., Rozek L.S., Taylor J.M.G., Light E., Chepeha D.B., Hébert J.R., Terrell J.E., Wolf G.T., Duffy S.A. (2013). Pretreatment Dietary Patterns, Weight Status, and Head and Neck Squamous Cell Carcinoma Prognosis. Am. J. Clin. Nutr..

[26] Fung T.T., Kashambwa R., Sato K., Chiuve S.E., Fuchs C.S., Wu K., Giovannucci E., Ogino S., Hu F.B., Meyerhardt J.A. (2014). Post Diagnosis Diet Quality and Colorectal Cancer Survival in Women. PLoS ONE.

[27] Kenfield S.A., DuPre N., Richman E.L., Stampfer M.J., Chan J.M., Giovannucci E.L. (2014). Mediterranean Diet and Prostate Cancer Risk and Mortality in the Health Professionals Follow-up Study. Eur. Urol..

[28] Kim E.H.J., Willett W.C., Fung T., Rosner B., Holmes M.D. (2011). Diet Quality Indices and Postmenopausal Breast Cancer Survival. Nutr. Cancer.

[29] Guinter M.A., McCullough M.L., Gapstur S.M., Campbell P.T. (2018). Associations of Pre- and Postdiagnosis Diet Quality with Risk of Mortality Among Men and Women with Colorectal Cancer. J. Clin. Oncol..

[30] Lee D.H., Fung T.T., Tabung F.K., Marinac C.R., Devore E.E., Rosner B.A., Ghobrial I.M., Colditz G.A., Giovannucci E.L., Birmann B.M. (2020). Prediagnosis Dietary Pattern and Survival in Patients with Multiple Myeloma. Int. J. Cancer.

[31] Crowder S.L., Sarma K.P., Mondul A.M., Chen Y.T., Li Z., Pepino M.Y., Zarins K.R., Wolf G.T., Rozek L.S., Arthur A.E. (2019). Pretreatment Dietary Patterns Are Associated with the Presence of Nutrition Impact Symptoms 1 Year after Diagnosis in Patients with Head and Neck Cancer. Cancer Epidemiol. Biomark. Prev..

[32] Izano M.A., Fung T.T., Chiuve S.S., Hu F.B., Holmes M.D. (2013). Are Diet Quality Scores after Breast Cancer Diagnosis Associated with Improved Breast Cancer Survival?. Nutr. Cancer.

[33] Al Ramadhani R.M., Nagle C.M., Ibiebele T.I., Grant P., Friedlander M., DeFazio A., Webb P.M., Ovarian Cancer Prognosis and Lifestyle Study Group (2021). Pre- and Post-Diagnosis Diet Quality and Ovarian Cancer Survival. Cancer Epidemiol. Biomark. Prev..

[34] Van Blarigan E.L., Zhang S., Ou F.-S., Venlo A., Ng K., Atreya C., Van Loon K., Niedzwiecki D., Giovannucci E., Wolfe E.G. (2020). Association of Diet Quality with Survival Among People With Metastatic Colorectal Cancer in the Cancer and Leukemia B and Southwest Oncology Group 80405 Trial. JAMA Netw. Open.

[35] Meyerhardt J.A., Niedzwiecki D., Hollis D., Saltz L.B., Hu F.B., Mayer R.J., Nelson H., Whittom R., Hantel A., Thomas J. (2007). Association of Dietary Patterns with Cancer Recurrence and Survival in Patients with Stage III Colon Cancer. JAMA.

[36] Sharma I., Roebothan B., Zhu Y., Woodrow J., Parfrey P.S., Mclaughlin J.R., Wang P.P. (2018). Hypothesis and Data-Driven Dietary Patterns and Colorectal Cancer Survival: Findings from Newfoundland and Labrador Colorectal Cancer Cohort. Nutr. J..

[37] Kwan M.L., Weltzien E., Kushi L.H., Castillo A., Slattery M.L., Caan B.J. (2009). Dietary Patterns and Breast Cancer Recurrence and Survival among Women with Early-Stage Breast Cancer. J. Clin. Oncol..

[38] Kroenke C.H., Fung T.T., Hu F.B., Holmes M.D. (2005). Dietary Patterns and Survival after Breast Cancer Diagnosis. J. Clin. Oncol..

[39] Lei Y., Ho S.C., Kwok C., Cheng A.C., Cheung K.L., Lee R., Yeo W. (2021). Dietary Pattern at 18-Month Post-Diagnosis and Outcomes of Breast Cancer Among Chinese Women with Early-Stage Breast Cancer. Cancer Manag. Res..

[40] Anyene I.C., Ergas I.J., Kwan M.L., Roh J.M., Ambrosone C.B., Kushi L.H., Cespedes Feliciano E.M. (2021). Plant-Based Dietary Patterns and Breast Cancer Recurrence and Survival in the Pathways Study. Nutrients.

[41] Ratjen I., Enderle J., Burmeister G., Koch M., Nöthlings U., Hampe J., Lieb W. (2021). Post-Diagnostic Reliance on Plant-Compared with Animal-Based Foods and All-Cause Mortality in Omnivorous Long-Term Colorectal Cancer Survivors. Am. J. Clin. Nutr..

[42] Ollberding N.J., Aschebrook-Kilfoy B., Caces D.B.D., Smith S.M., Weisenburger D.D., Chiu B.C.-H. (2013). Dietary Intake of Fruits and Vegetables and Overall Survival in Non-Hodgkin Lymphoma. Leuk. Lymphoma.

[43] van Zutphen M., Boshuizen H.C., Kenkhuis M.-F., Wesselink E., Geijsen A.J.M.R., de Wilt J.H.W., van Halteren H.K., Spillenaar Bilgen E.J., Keulen E.T.P., Janssen-Heijnen M.L.G. (2021). Lifestyle after Colorectal Cancer Diagnosis in Relation to Recurrence and All-Cause Mortality. Am. J. Clin. Nutr..

[44] Song R., Petimar J., Wang M., Tabung F.K., Song M., Liu L., Lee D.H., Giovannucci E.L., Zhang X., Smith-Warner S.A. (2021). Adherence to the World Cancer Research Fund/American Institute for Cancer Research Cancer Prevention Recommendations and Colorectal Cancer Survival. Cancer Epidemiol. Biomark. Prev..

[45] Romaguera D., Ward H., Wark P.A., Vergnaud A.-C., Peeters P.H., van Gils C.H., Ferrari P., Fedirko V., Jenab M., Boutron-Ruault M.-C. (2015). Pre-Diagnostic Concordance with the WCRF/AICR Guidelines and Survival in European Colorectal Cancer Patients: A Cohort Study. BMC Med..

[46] Inoue-Choi M., Lazovich D., Prizment A.E., Robien K. (2013). Adherence to the World Cancer Research Fund/American Institute for Cancer Research Recommendations for Cancer Prevention Is Associated with Better Health-Related Quality of Life among Elderly Female Cancer Survivors. J. Clin. Oncol..

[47] Di Maso M., Dal Maso L., Augustin L.S.A., Puppo A., Falcini F., Stocco C., Mattioli V., Serraino D., Polesel J. (2020). Adherence to the Mediterranean Diet and Mortality after Breast Cancer. Nutrients.

[48] Ratjen I., Schafmayer C., di Giuseppe R., Waniek S., Plachta-Danielzik S., Koch M., Nöthlings U., Hampe J., Schlesinger S., Lieb W. (2017). Postdiagnostic Mediterranean and Healthy Nordic Dietary Patterns Are Inversely Associated with All-Cause Mortality in Long-Term Colorectal Cancer Survivors. J. Nutr..

[49] Zheng J., Tabung F.K., Zhang J., Murphy E.A., Shivappa N., Ockene J.K., Caan B., Kroenke C.H., Hébert J.R., Steck S.E. (2020). Post-Cancer Diagnosis Dietary Inflammatory Potential Is Associated with Survival among Women Diagnosed with Colorectal Cancer in the Women’s Health Initiative. Eur. J. Nutr..

[50] Zheng J., Tabung F.K., Zhang J., Liese A.D., Shivappa N., Ockene J.K., Caan B., Kroenke C.H., Hébert J.R., Steck S.E. (2018). Association between Post-Cancer Diagnosis Dietary Inflammatory Potential and Mortality among Invasive Breast Cancer Survivors in the Women’s Health Initiative. Cancer Epidemiol. Biomark. Prev..

[51] Jang H., Chung M.S., Kang S.S., Park Y. (2018). Association between the Dietary Inflammatory Index and Risk for Cancer Recurrence and Mortality among Patients with Breast Cancer. Nutrients.

[52] Zucchetto A., Gini A., Shivappa N., Hébert J.R., Stocco C., Dal Maso L., Birri S., Serraino D., Polesel J. (2016). Dietary Inflammatory Index and Prostate Cancer Survival. Int. J. Cancer.

[53] Yuan C., Bao Y., Sato K., Nimptsch K., Song M., Brand-Miller J.C., Morales-Oyarvide V., Zoltick E.S., Keum N., Wolpin B.M. (2017). Influence of Dietary Insulin Scores on Survival in Colorectal Cancer Patients. Br. J. Cancer.

[54] Tabung F.K., Noonan A., Lee D.H., Song M., Clinton S.K., Spakowicz D., Wu K., Cheng E., Meyerhardt J.A., Fuchs C.S. (2020). Post-Diagnosis Dietary Insulinemic Potential and Survival Outcomes among Colorectal Cancer Patients. BMC Cancer.

[55] Wang T., Farvid M.S., Kang J.H., Holmes M.D., Rosner B.A., Tamimi R.M., Willett W.C., Eliassen A.H. (2021). Diabetes Risk Reduction Diet and Survival after Breast Cancer Diagnosis. Cancer Res..

[56] McCullough M.L., Gapstur S.M., Shah R., Campbell P.T., Wang Y., Doyle C., Gaudet M.M. (2016). Pre- and Postdiagnostic Diet in Relation to Mortality among Breast Cancer Survivors in the CPS-II Nutrition Cohort. Cancer Causes Control.

[57] Ferronha I., Castro C., Carreira H., Bento M.J., Carvalho I., Peleteiro B., Lunet N. (2012). Prediagnosis Lifestyle Exposures and Survival of Gastric Cancer Patients: A Cohort Study from Portugal. Br. J. Cancer.

[58] Palesh O., Kamen C., Sharp S., Golden A., Neri E., Spiegel D., Koopman C. (2018). Physical Activity and Survival in Women With Advanced Breast Cancer. Cancer Nurs..

[59] Patel A.V., Bernstein L., Deka A., Feigelson H.S., Campbell P.T., Gapstur S.M., Colditz G.A., Thun M.J. (2010). Leisure Time Spent Sitting in Relation to Total Mortality in a Prospective Cohort of US Adults. Am. J. Epidemiol..

[60] Hamer M., de Oliveira C., Demakakos P. (2014). Non-Exercise Physical Activity and Survival: English Longitudinal Study of Ageing. Am. J. Prev. Med..

[61] Watts E.L., Matthews C.E., Freeman J.R., Gorzelitz J.S., Hong H.G., Liao L.M., McClain K.M., Saint-Maurice P.F., Shiroma E.J., Moore S.C. (2022). Association of Leisure Time Physical Activity Types and Risks of All-Cause, Cardiovascular, and Cancer Mortality Among Older Adults. JAMA Netw. Open.

[62] Hansen J.M., Nagle C.M., Ibiebele T.I., Grant P.T., Obermair A., Friedlander M.L., DeFazio A., Webb P.M., Ovarian Cancer Prognosis and Lifestyle Study Group (2020). A Healthy Lifestyle and Survival among Women with Ovarian Cancer. Int. J. Cancer.

[63] Friedenreich C.M., Wang Q., Neilson H.K., Kopciuk K.A., McGregor S.E., Courneya K.S. (2016). Physical Activity and Survival After Prostate Cancer. Eur. Urol..

[64] Di Maso M., Augustin L.S.A., Toffolutti F., Stocco C., Dal Maso L., Jenkins D.J.A., Fleshner N.E., Serraino D., Polesel J. (2021). Adherence to Mediterranean Diet, Physical Activity and Survival after Prostate Cancer Diagnosis. Nutrients.

[65] Walter V., Jansen L., Knebel P., Chang-Claude J., Hoffmeister M., Brenner H. (2017). Physical Activity and Survival of Colorectal Cancer Patients: Population-Based Study from Germany. Int. J. Cancer.

[66] Jee Y., Kim Y., Jee S.H., Ryu M. (2018). Exercise and Cancer Mortality in Korean Men and Women: A Prospective Cohort Study. BMC Public Health.

[67] Cannioto R.A., Hutson A., Dighe S., McCann W., McCann S.E., Zirpoli G.R., Barlow W., Kelly K.M., DeNysschen C.A., Hershman D.L. (2021). Physical Activity Before, During, and After Chemotherapy for High-Risk Breast Cancer: Relationships With Survival. J. Natl. Cancer Inst..

[68] Ammitzbøll G., Søgaard K., Karlsen R.V., Tjønneland A., Johansen C., Frederiksen K., Bidstrup P. (2016). Physical Activity and Survival in Breast Cancer. Eur. J. Cancer.

[69] Mok Y., Jeon C., Lee G.J., Jee S.H. (2016). Physical Activity Level and Colorectal Cancer Mortality. Asia Pac. J. Public Health.

[70] Cao C., Friedenreich C.M., Yang L. (2022). Association of Daily Sitting Time and Leisure-Time Physical Activity With Survival Among US Cancer Survivors. JAMA Oncol..

[71] Bian Z., Zhang R., Yuan S., Fan R., Wang L., Larsson S.C., Theodoratou E., Zhu Y., Wu S., Ding Y. (2024). Healthy Lifestyle and Cancer Survival: A Multinational Cohort Study. Int. J. Cancer.

[72] Johnsson A., Broberg P., Krüger U., Johnsson A., Tornberg Å.B., Olsson H. (2019). Physical Activity and Survival Following Breast Cancer. Eur. J. Cancer Care.

[73] Choi J., Park J.-Y., Kim J.-E., Lee M., Lee K., Lee J.-K., Kang D., Shin A., Choi J.-Y. (2023). Impact of Pre- and Post-Diagnosis Physical Activity on the Mortality of Patients with Cancer: Results from the Health Examinees-G Study in Korea. Cancer Med..

[74] Thorsen L., Courneya K.S., Steene-Johannessen J., Gran J.M., Haugnes H.S., Negaard H.F.S., Kiserud C.E., Fosså S.D. (2023). Association of Physical Activity with Overall Mortality among Long-Term Testicular Cancer Survivors: A Longitudinal Study. Int. J. Cancer.

[75] Sheikh M., Mukeriya A., Shangina O., Brennan P., Zaridze D. (2021). Postdiagnosis Smoking Cessation and Reduced Risk for Lung Cancer Progression and Mortality: A Prospective Cohort Study. Ann. Intern. Med..

[76] Gemine R.E., Ghosal R., Collier G., Parry D., Campbell I., Davies G., Davies K., Lewis K.E., LungCast Investigators (2019). Longitudinal Study to Assess Impact of Smoking at Diagnosis and Quitting on 1-Year Survival for People with Non-Small Cell Lung Cancer. Lung Cancer.

[77] Koshiaris C., Aveyard P., Oke J., Ryan R., Szatkowski L., Stevens R., Farley A. (2017). Smoking Cessation and Survival in Lung, Upper Aero-Digestive Tract and Bladder Cancer: Cohort Study. Br. J. Cancer.

[78] Ferketich A.K., Niland J.C., Mamet R., Zornosa C., D’Amico T.A., Ettinger D.S., Kalemkerian G.P., Pisters K.M., Reid M.E., Otterson G.A. (2013). Smoking Status and Survival in the National Comprehensive Cancer Network Non-Small Cell Lung Cancer Cohort. Cancer.

[79] Sheikh M., Mukeriya A., Zahed H., Feng X., Robbins H.A., Shangina O., Matveev V., Brennan P., Zaridze D. (2023). Smoking Cessation After Diagnosis of Kidney Cancer Is Associated with Reduced Risk of Mortality and Cancer Progression: A Prospective Cohort Study. J. Clin. Oncol..

[80] Tse L.A., Lin X., Li W., Qiu H., Chan C.K., Wang F., Yu I.T.-S., Leung C.C. (2018). Smoking Cessation Sharply Reduced Lung Cancer Mortality in a Historical Cohort of 3185 Chinese Silicotic Workers from 1981 to 2014. Br. J. Cancer.

[81] Chen J., Qi Y., Wampfler J.A., Jatoi A., Garces Y.I., Busta A.J., Mandrekar S.J., Yang P. (2012). Effect of Cigarette Smoking on Quality of Life in Small Cell Lung Cancer Patients. Eur. J. Cancer.

[82] Chang L.C., Loh E.W., Tsai Y.W., Chiou S.T., Chen L.K. (2014). Clinical Benefits of Smoking Cessation in Reducing All-Cause and Disease-Specific Mortality among Older People in Taiwan: A 10-Year Nationwide Retrospective Cohort Study. Eur. Geriatr. Med..

[83] Kenfield S.A., Stampfer M.J., Chan J.M., Giovannucci E. (2011). Smoking and Prostate Cancer Survival and Recurrence. JAMA.

[84] Heberg J., Simonsen M.K., Thomsen T., Zoffmann V., Danielsen A.K. (2020). Smoking Cessation Prolongs Survival in Female Cancer Survivors-the Danish Nurse Cohort. Eur. J. Oncol. Nurs..

[85] Japuntich S.J., Kumar P., Pendergast J.F., Juarez Caballero G.Y., Malin J.L., Wallace R.B., Chrischilles E.A., Keating N.L., Park E.R. (2018). Smoking Status and Survival Among a National Cohort of Lung and Colorectal Cancer Patients. Nicotine Tob. Res..

[86] Jang D., Choe S., Park J.W., Jeong S.-Y., Shin A. (2020). Smoking Status before and after Colorectal Cancer Diagnosis and Mortality in Korean Men: A Population-Based Cohort Study. Cancer Med..

[87] Rades D., Setter C., Schild S.E., Dunst J. (2008). Effect of Smoking during Radiotherapy, Respiratory Insufficiency, and Hemoglobin Levels on Outcome in Patients Irradiated for Non-Small-Cell Lung Cancer. Int. J. Radiat. Oncol. Biol. Phys..

[88] Dobson Amato K.A., Hyland A., Reed R., Mahoney M.C., Marshall J., Giovino G., Bansal-Travers M., Ochs-Balcom H.M., Zevon M.A., Cummings K.M. (2015). Tobacco Cessation May Improve Lung Cancer Patient Survival. J. Thorac. Oncol..

[89] Tao L., Wang R., Gao Y.-T., Yuan J.-M. (2013). Impact of Postdiagnosis Smoking on Long-Term Survival of Cancer Patients: The Shanghai Cohort Study. Cancer Epidemiol. Biomark. Prev..

[90] Linhas A.R.D., Dias M.C.P., Barroso A.M.P. (2018). Smoking Cessation before Initiation of Chemotherapy in Metastatic Non-Small Cell Lung Cancer: Influence on Prognosis. J. Bras. Pneumol..

[91] Jamrozik K., McLaughlin D., McCaul K., Almeida O.P., Wong K.Y., Vagenas D., Dobson A. (2011). Women Who Smoke like Men Die like Men Who Smoke: Findings from Two Australian Cohort Studies. Tob. Control.

[92] Frost G., Darnton A., Harding A.-H. (2011). The Effect of Smoking on the Risk of Lung Cancer Mortality for Asbestos Workers in Great Britain (1971–2005). Ann. Occup. Hyg..

[93] Jung E.-J., Shin A., Park S.K., Ma S.-H., Cho I.-S., Park B., Lee E.-H., Chang S.-H., Shin H.-R., Kang D. (2012). Alcohol Consumption and Mortality in the Korean Multi-Center Cancer Cohort Study. J. Prev. Med. Public Health.

[94] Minami Y., Kanemura S., Kawai M., Nishino Y., Tada H., Miyashita M., Ishida T., Kakugawa Y. (2019). Alcohol Consumption and Survival after Breast Cancer Diagnosis in Japanese Women: A Prospective Patient Cohort Study. PLoS ONE.

[95] Hermans K.E.P.E., van den Brandt P.A., Loef C., Jansen R.L.H., Schouten L.J. (2021). Alcohol Consumption, Cigarette Smoking and Cancer of Unknown Primary Risk: Results from the Netherlands Cohort Study. Int. J. Cancer.

[96] Vrieling A., Buck K., Heinz J., Obi N., Benner A., Flesch-Janys D., Chang-Claude J. (2012). Pre-Diagnostic Alcohol Consumption and Postmenopausal Breast Cancer Survival: A Prospective Patient Cohort Study. Breast Cancer Res. Treat..

[97] Tamakoshi A., Nakamura K., Ukawa S., Okada E., Hirata M., Nagai A., Matsuda K., Kamatani Y., Muto K., Kiyohara Y. (2017). Characteristics and Prognosis of Japanese Colorectal Cancer Patients: The BioBank Japan Project. J. Epidemiol..

[98] Farris M.S., Courneya K.S., Kopciuk K.A., McGregor S.E., Friedenreich C.M. (2018). Post-Diagnosis Alcohol Intake and Prostate Cancer Survival: A Population-Based Cohort Study. Int. J. Cancer.

[99] Yang B., Gapstur S.M., Newton C.C., Jacobs E.J., Campbell P.T. (2017). Alcohol Intake and Mortality among Survivors of Colorectal Cancer: The Cancer Prevention Study II Nutrition Cohort. Cancer.

[100] Walter V., Jansen L., Ulrich A., Roth W., Bläker H., Chang-Claude J., Hoffmeister M., Brenner H. (2016). Alcohol Consumption and Survival of Colorectal Cancer Patients: A Population-Based Study from Germany. Am. J. Clin. Nutr..

[101] Koyama S., Tabuchi T., Morishima T., Miyashiro I. (2024). Alcohol Consumption and 10-Year Mortality in Oral and Pharyngeal Cancer. Cancer Epidemiol..

[102] Beynon R.A., Lang S., Schimansky S., Penfold C.M., Waylen A., Thomas S.J., Pawlita M., Waterboer T., Martin R.M., May M. (2018). Tobacco Smoking and Alcohol Drinking at Diagnosis of Head and Neck Cancer and All-Cause Mortality: Results from Head and Neck 5000, a Prospective Observational Cohort of People with Head and Neck Cancer. Int. J. Cancer.

[103] Ramadas K., Sauvaget C., Thomas G., Fayette J.-M., Thara S., Sankaranarayanan R. (2010). Effect of Tobacco Chewing, Tobacco Smoking and Alcohol on All-Cause and Cancer Mortality: A Cohort Study from Trivandrum, India. Cancer Epidemiol..

[104] Denissoff A., Huusko T., Ventelä S., Niemelä S., Routila J. (2022). Exposure to Alcohol and Overall Survival in Head and Neck Cancer: A Regional Cohort Study. Head. Neck.

[105] Din N., Allen I.E., Satariano W.A., Demb J., Braithwaite D. (2016). Alcohol Consumption and Mortality after Breast Cancer Diagnosis: The Health and Functioning in Women Study. Breast Dis..

[106] Yi S.-W., Sull J.W., Linton J.A., Nam C.M., Ohrr H. (2010). Alcohol Consumption and Digestive Cancer Mortality in Koreans: The Kangwha Cohort Study. J. Epidemiol..

[107] Jayasekara H., English D.R., Haydon A., Hodge A.M., Lynch B.M., Rosty C., Williamson E.J., Clendenning M., Southey M.C., Jenkins M.A. (2018). Associations of Alcohol Intake, Smoking, Physical Activity and Obesity with Survival Following Colorectal Cancer Diagnosis by Stage, Anatomic Site and Tumor Molecular Subtype. Int. J. Cancer.

[108] Jankhotkaew J., Bundhamcharoen K., Suphanchaimat R., Waleewong O., Chaiyasong S., Markchang K., Wongworachate C., Vathesatogkit P., Sritara P. (2020). Associations between Alcohol Consumption Trajectory and Deaths Due to Cancer, Cardiovascular Diseases and All-Cause Mortality: A 30-Year Follow-up Cohort Study in Thailand. BMJ Open.

[109] McCain R.S., McManus D.T., McQuaid S., James J.A., Salto-Tellez M., Reid N.B., Craig S., Chisambo C., Bingham V., McCarron E. (2020). Alcohol Intake, Tobacco Smoking, and Esophageal Adenocarcinoma Survival: A Molecular Pathology Epidemiology Cohort Study. Cancer Causes Control..

[110] Zeinomar N., Qin B., Amin S., Lin Y., Xu B., Chanumolu D., Omene C.O., Pawlish K.S., Demissie K., Ambrosone C.B. (2023). Association of Cigarette Smoking and Alcohol Consumption With Subsequent Mortality Among Black Breast Cancer Survivors in New Jersey. JAMA Netw. Open.

[111] Mayne S.T., Cartmel B., Kirsh V., Goodwin W.J. (2009). Alcohol and Tobacco Use Prediagnosis and Postdiagnosis, and Survival in a Cohort of Patients with Early Stage Cancers of the Oral Cavity, Pharynx, and Larynx. Cancer Epidemiol. Biomarkers Prev..

