# Supplementary material for: Impact of Lifestyle Modifications on Cancer Mortality: A Systematic Review and Meta-Analysis
Source: Medicina (Kaunas). 2025 Feb 10;61(2):307. doi: 10.3390/medicina61020307 (PMC11857246; doi:10.3390/medicina61020307)
Supplement: Supplementary file 1 [file medicina-61-00307-s001.zip › medicina-3441616-supplementary.pdf]

Table S1: Characteristics of studies associated with Dietary changes

ID.	Study	Country	Study Design	Cancer Type	Number of Participants	Follow up Duration	Intervention	main outcomes
1.	Park et al. 2022	USA	Cohort study	All cancer types	6,370	10.7 years	Diet	A post-diagnostic high-quality diet was associated with a reduced all-cause and cancer mortality among adult cancer survivors, with a risk reduction similar to that among participants with no cancer.
2.	Anyene et al. 2021	USA	Cohort study	Breast cancer	3,646	9.51 years	Diet	A healthful plant-based dietary pattern may decrease non-breast-cancer mortality, whereas an unhealthful plant-based dietary pattern increase risk. Therefore, quality of plant foods should be considered to derive a healthful dietary pattern. Healthful plant-based diets may improve overall survival in breast cancer survivors.
3.	Di Maso et al. 2021	Italy	Case-control	Prostate cancer	777	10 years	Diet	No association was observed regarding Mediterranean diet being associated with reduced cancer-specific mortality, support the beneficial impact of pre-diagnostic adherence to the Mediterranean diet and physical activity on overall survival after prostate cancer diagnosis, mainly due to lower non-PCa-specific mortality.

4.	Ergas et al. 2021	USA	Cohort study	Breast cancer	3,660	40.888 person years	Diet	There was an indication in the study that a r eduction in the risk of non-breast cancer-specific and all-cause mortality was associated with the ACS, aMED, DASH, and HEI. However, dietary quality indices were not related to breast cancer-specific outcomes.
5.	Lei et al. 2021	China	Cohort study	Breast cancer	1,226	54.1 months	Diet	Neither dietary pattern, the Western dietary pattern or the Healthy dietary pattern, was associated with the risk of breast cancer recurrence, all-causes death, or death from breast cancer.
6.	Ratjen et al. 2021	Germany	Cohort study	Colorectal cancer	1,404	7 years	Diet	The overall plant-based diet index was significantly inversely associated with all-cause mortality. In subgroup analysis, the tendency towards a positive association between the unhealthful plant-based diet with mortality was confined to less physically active individuals.
7.	Van Zutphen et al. 2021	Netherlands	Cohort study	Colorectal cancer	1,425	2.6 (for cancer recurrence) 4.4 (for mortality	Diet	Although healthy lifestyle after diagnosis was not associated with CRC recurrence in patients with stage I-III CRC, it tended to be related to reduced risk of all-cause mortality.

8.	Al Ramadhani et al. 2021	Australia	Cohort study	Ovarian cancer	1,153	4.4 years	Diet	In this cohort of women with ovarian cancer, higher pre- and post-diagnosis diet quality was not associated with improved survival.
9.	Song et al. 2021	USA	Cohort study	Colorectal cancer	1,491	7.92 years	Diet	This post-diagnostic WCRF/AICR diet score has not shown a statistically significant association with either colorectal cancer-specific or overall mortality.
10.	Wang 2021	USA	Cohort study	Breast cancer	8,482	14 years	Diet	The study findings provide evidence that greater adherence to a DRRD is associated with reduced mortality after breast cancer diagnosis.
11.	Hansen 2020	Australia	OPAL study	Ovarian cancer	958	Prediagnosis - 3.9 years, Postdiagnosis- 3.5 years	Diet	There was no association between pre diagnosis physical activity, alcohol or diet quality score and survival and There was no significant association between BMI or diet quality postdiagnosis and all-cause mortality.
**12.**	Lee 2020	USA	Cohort study	Multiple myeloma	423	3.5	Diet	These consistent findings for multiple dietary patterns provide the first evidence that Multiple Myeloma patients with healthier pre-diagnosis dietary habits may have longer survival than less healthy dieting patients.

13.	Tabung 2020	USA	Cohort study	Colorectal cancer	1,718	9.9 years	Diet	Findings suggest that a hyper insulinemic dietary pattern after diagnosis of colorectal cancer is associated with poorer survival.
14.	Van Blarigan et al. 2020	USA	Cohort study	Colorectal cancer	1,284	73 months	Diet	In the prospective analysis among patients with metastatic colorectal cancer, diet quality as assessed at the initiation of first-line treatment for metastatic disease was not associated with overall survival.
**15.**	Wang 2020	China	Cohort study	Breast cancer	3,450	5 years	Diet	Higher post-diagnosis adherence to the CHFP and DASH dietary guidelines was associated with reduced risk of both overall death and breast cancer-specific recurrence or death among long-term breast cancer survivors.
16.	Di Maso et al. 2020	Italy	Case-control	Breast cancer	1,453	12.6 years	Diet	pre-diagnosis consumption of more diet that in line with The Mediterranean dietary pattern in female breast cancer patients may have better prognosis post-diagnosis than in those with less consumption.
17.	Zheng 2020	USA	Cohort study	Colorectal cancer	463	11.6 years	Diet	Consumption of an anti-inflammatory dietary pattern and supplements post CRC diagnosis is associated with lower all-cause mortality in postmenopausal women.

18.	Luo et al. 2020	China	Cohort study	Hepatocellular carcinoma	887	797 days	Diet	Findings suggest that higher adherence to the 2016 Dietary Guidelines for Chinese is associated with reduced risk of both all-cause and hepatocellular carcinoma-specific death among HCC patients
19.	Crowder 2019	USA	Cohort study	Head and Neck cancer	336	1 year	Diet	A prudent diet consumed before treatment may be associated with a reduced risk of chronic nutrition impact symptoms burden among head and neck cancer survivors.
20.	Karavasiloglou et al. 2019	USA	Cohort study	Breast cancer or gynecological cancer	230	15 years	Diet	Diet quality, measured by the HEI, was inversely associated with post-diagnostic mortality in female survivors.
21.	Jang 2018	Korea	Cohort study	Breast cancer	511	69 months	Diet	Current findings showed that anti-inflammatory diet may reduce recurrence and overall mortality in patients with breast cancer, particularly in cancers presenting with factors of poor prognosis, such as younger age, premenopausal status, obesity, HR+ status of the tumor, tumor size >2 cm, and presence of lymph node metastasis.
22.	Sun 2018	USA	Cohort study	Breast cancer	2,295	12 years	Diet	Women who decreased diet quality, defined as a ≥15% decrease in HEI-2010 score, had a higher risk of death due to breast cancer.

								However, increased diet quality, defined as a ≥15% increase in HEI-2010 score, was not significantly associated with lower risk of death.
23.	Zheng et al. 2018	USA	Cohort study	Breast cancer	2,150	13,3 years	Diet	Energy-adjusted dietary inflammatory index scores were associated with lower Cardio vascular mortality but not with breast cancer specific or all-cause mortality.
24.	Guinter et al. 2018	USA	Cohort study	Colorectal cancer	Pre-diagnosis: 2671, Post-diagnosis:1, 321	Pre-diagnosis: 6.5 (among participants who died); 11.4 (among participants who were alive at the end of the study); post-diagnosis: 6.4 (among participants who died);	Diet	The pre-diagnosis score of ACS was inversely related to all-cause and CRC-specific mortality, whereas the Western diet score was associated with a higher all-cause mortality. The ACS-score post-diagnosis diet had a lower risk for all-cause and CRC-specific mortality, DASH score inversely associated with all and CRC-specific mortality, and prudent score inversely with all-cause mortality. Among those with low diet quality pre-diagnosis, higher DASH and prudent scores from pre-diagnosis to post-diagnosis were inversely associated with CRC-specific mortality.

						13.5 (among participants who were alive at the		
25.	Sharma et al. 2018	Canada	Cohort study	Colorectal cancer	532	6.27 years	Diet	Estimates of overall mortality and combined mortality, recurrence or metastasis differed by dietary patterns; aMED, RFS, Processed meat pattern, Prudent vegetable pattern and High sugar pattern, due to differences in the derivation basis of each pattern.
26.	Deshmukh et al. 2018	USA	Cohort study	All cancer types	1,191	17.2 years	Diet	Overall, high-quality food intake, as assessed by high HEI scores, may be protective against death among cancer survivors.
27.	Ratjen et al. 2017	Germany	Cohort study	Colorectal cancer	1,404	7 years	Diet	Greater adherences to the Mediterranean diet and to the healthy Nordic diet after CRC diagnosis are associated with better overall survival in long-term CRC survivors.
28.	Yuan 2017	USA	Cohort study	Colorectal cancer	2,006	12.7 years	Diet	Higher dietary insulin scores after Colorectal cancer diagnosis were associated with statistically significant increase in Colorectal cancer-specific and overall mortality.

29.	Jacobs et al. 2016	USA	Cohort study	Colorectal cancer	4,204	6 years	Diet	The aMED score was associated with lower mortality only in African-American women not men (1 of 5 ethnic groups studied).
30.	Zucchetto et al. 2016	Italy	Case-control	Prostate cancer	726	12.7 years	Diet	Dietary Inflammatory Index scores were not significantly associated with all-cause mortality of prostate cancer patients
31.	McCullough et al. 2016	USA	Cohort study	Breast cancer	4,452	9.8 years	Diet	Diets consistent with guidelines for cancer prevention were not associated with breast cancer-specific mortality neither pre or post diagnostic
32.	Romaguera 2015	Europe	Cohort study	Colorectal cancer	3,292	4.2 years	Diet	The better concordance with the WCRF/AICR recommendations on diet, physical activity, and body fatness before diagnosis of CRC was associated with improved cancer-specific survival among CRC patients.
33.	Fung et al. 2014	USA	Cohort study	Colorectal cancer	1,201	11.2 years	Diet	Higher AHEI-2010 score might be associated with lower overall mortality
34.	Kenfield et al. 2014	USA	Cohort study	Prostate cancer	4,538	9.1 years	Diet	Higher aMED scores was not associated with a decrease in cancer-specific mortality.
35.	Thomson et al. 2014	USA	Cohort study	Ovarian cancer	636	17 years	Diet	The higher the quality of diet, the lower all-cause mortality after ovarian cancer but not cancer-specific mortality.
36.	Pelser et al. 2014	USA	Cohort study	Colon cancer,	5,727	5 years	Diet	A higher diet quality, as assessed by the HEI-2005, was associated with lower

				Rectal cancer				all-cause mortality, prior to colon and rectal cancer, but not with cancer-specific mortality.
37.	Arthur et al. 2013	USA	Cohort study	Head and neck squamous cell carcinoma	542	6 years	Diet	Intake of a diet high in vegetables, fruit, fish, poultry, and whole grains and overweight status before head and neck cancer diagnosis are associated with a better prognosis.
38.	Inoue-Choi et al. 2013	USA	Cohort study	Breast, colorectal, gynecologic and other cancers	2,017	5.4 years	Diet	We found that elderly female cancer survivors who met a greater number of the WCRF/AICR recommendations had better physical and mental health-related quality of life.
39.	Izano et al. 2013	USA	Cohort	Breast cancer	4,103	112 months	Diet	The diet scores are not found to have association with mortality due to breast cancer significantly.
40.	Ollberding et al. 2013	USA	Case-control	Non-Hodgkin lymphoma	301	8.2 years	Diet	The data suggests that pre-diagnostic fruit and vegetable consumption is not associated with overall survival in lymphoma patients
41.	Vrieling et al. 2013	Germany	Cohort study	Breast cancer	2,522	5.5 years	Diet	Greater consumption of a Western or 'unhealthy' dietary pattern before diagnosis may result in an increased non-breast cancer mortality as well as overall mortality risk

42.	Ferronha et al. 2012	Portugal	Cohort study	Gastric cancer	568	6.6 (for those alive at end) 1.2 (for those who died)	Diet	lifestyles including dietary patterns before the diagnosis have negligible impact on the survival of gastric cancer patients.
43.	Kim et al. 2011	USA	Cohort study	Breast cancer	2,729	Follow-up time not shown; diagnosed between 1978 and 1998, followed through 2004	Diet	Our results suggested that a higher quality diet after diagnosis of breast cancer doesn't play a significant role in alter ing mortality from breast cancer.
44.	Kwan et al. 2009	USA	Cohort study	Breast cancer	1,901	5.93 years	Diet	Neither dietary pattern was associated with risk of breast cancer recurrence or death from breast cancer
45.	Meyerhardt et al. 2007	USA	Cohort study	Colon cancer	1,009	5.3 years	Diet	Higher intake of a Western dietary pattern may be associated with a higher risk of recurrence and mortality among patients
46.	Kroenke et al. 2005	USA	Cohort study	Breast cancer	2,619	9 years	Diet	A higher intake of the prudent pattern and a lower intake of the Western pattern may protect against mortality from causes

	unrelated to breast cancer but not breast cancer related death

Table S2: Characteristics of studies associated with Physical Activity:

	Study ID	Location	Study Design	Cancer Type	Participants (N)	Follow up duration (Median)		Outcomes
1.	Palesh O et al 2018	US	Cohort	Breast	103	5.4 years	Physical activity	Women with advanced breast cancer who engaged in at least one hour of physical activity per day at baseline had a higher likelihood of survival compared to those who exercised for less than one hour daily
2.	Patel AV et al 2010	US	Cohort	Mixed	69776	14 years	Physical activity	Prolonged sitting time was linked to a higher risk of cancer mortality in women. Total physical activity was modestly linked to lower cancer mortality in women.
3.	Hamer M 2014	UK	cohort	mixed	7008	7.8 years	Physical activity	Vigorous physical activity was linked to a reduced risk of cancer mortality, not mild physical activity.
4.	Ueshima K et al 2009	Japan	Cohort	Mixed	10385	8 years	Physical activity	The relationship between physical activity and cancer mortality remained unclear.
5.	Watts EL et al 2022	US	Cohort	Mixed	272550	12.4 years	Physical activity	A cohort study of older adults found variations in mortality risk depending on the type of leisure activities, but significant associations were observed between

								engaging in 7.5 to less than 15 MET hours per week of any activity and reduced mortality risk due to cancers.
6.	Hansen JM et al 2020	Australia	Cohort	Ovarian Ca	678	Not specified	Physical activity	Higher physical activity after diagnosis was associated with better survival among ovarian cancer patients
7.	Van Blarigan EL et al 2018	US	Cohort	Colon cancer	992	7 years	Physical activity	Engaging in regular physical activity after a stage III colon cancer diagnosis was linked to improved survival.
8.	Friedenreich CM et al 2016	Canada	Cohort	prostrate	830	15.5 years	Physical activity	One 17-year prostate cancer survival study found that men who survived at least 2 years and were more physically active after diagnosis, or engaged in greater recreational physical activity both before and after diagnosis had longer survival. Recreational physical activity after diagnosis was associated with a lower risk of prostate cancer-specific death.
9.	Di Maso M et al 2021	Italy	Cohort	prostrate	777	10 years	Physical activity	high physical activity was not significantly associated with overall survival in prostrate cancer patients
10.	Walter V et al 2017	Germany	Cohort	Colon cancer	3121	4.8 years	Physical activity	Physical activity prior to diagnosis was linked to a better prognosis in colorectal cancer (CRC). These associations may be limited to specific types of activities or influenced by the presence or absence of metastatic disease.
11.	Jee Y et al 2018	Korea	cohort	mixed	3,03,428	15.3 years	Physical activity	There was a curvilinear relationship between weekly exercise duration and cancer mortality. The lowest risk was identified at low to moderate levels of exercise. This pattern was evident for esophageal, liver, lung, and colorectal cancer mortality in men, as well as for all-cause, all-cancer, and lung cancer mortality in women.

12.	Cannioto RA et al 2021	US	Cohort	Breast	1340	2 years	Physical activity	Adhering to the minimum physical activity guidelines both prior to diagnosis and following treatment seems to be linked to a statistically significant reduction in the risk of recurrence and mortality among breast cancer patients.
13.	Ammitzbøll G et al 2016	Denmark	Cohort	Breast	959	Not specified	Physical activity	Engaging in 2.5 hours or more of brisk walking each week after a breast cancer diagnosis may lower mortality by as much as 32% compared to those who engage in low levels of exercise. Additionally, participating in regular physical activity can reduce mortality by 44% compared to non-participants.
14.	Mok Y et al 2016	Korea	Cohort	colo rectal ca	226089	Not specified	Physical activity	The link between high-intensity physical activity and colon cancer survival in men approached statistical significance for those exceeding 17.5 MET-hours per week. Engaging in regular physical activity may offer benefits for survival among colon cancer patients.
15.	Cao C et al 2022	US	Cohort	Mixed	1535	4.5 years	Physical activity	In this cohort study, performed on a representative sample of US cancer survivors, one observed that prolonged sitting combined with low levels of physical activity was extremely common and carried the highest risk of death from all causes and cancer.
16.	Friedenreich CM et al 2020	Canada	Cohort	Endometrial Cancer	425	3.4 years	Physical activity	Recreational physical activity, especially postdiagnosis, is associated with improved survival in survivors of endometrial cancer.
17.	Bian Z et al 2024	UK	Cohort	Mixed	37095	Not specified	Physical activity	Sufficient physical activity was significantly associated with a decreased risk of all-cause mortality among cancer

								survivors, with an adjusted HR of 0.90 (95% CI: 0.85, 0.94).
18.	Johnsson A et al 2019	Sweden	Cohort	Breast	847	Not specified	Physical activity	After a breast cancer diagnosis, it is important to promote physical activity, which is a modifiable risk factor, especially in post-menopausal women with breast cancer.
19.	Choi J et al 2023	Korea	Cohort	colo rectal ca	3008	12.3 years	Physical activity	Physical activity, particularly vigorous-intensity activities, walking, climbing, sports, and engaging in more than two activities pre-diagnosis, as well as performing multiple activities post-diagnosis, were significantly associated with lower all-cause and cancer-specific mortality among Korean cancer patients.
20.	Thorsen L et al 2023	Norway	Cohort	Testicula r Ca	1437	20 years	Physical activity	Regular and sustained physical activity during long-term survivorship in testicular cancer survivors was associated with at least a 50% reduction in overall mortality risk, with Actives showing a significantly lower mortality compared to Inactives.

Table S3: Characteristics of studies associated with Smoking Cessation:

	Study ID	Location	Study Design	Cancer Type	Participants (N)	Follow up duration (Median)	Intervention type	Outcome
1.	Sheikh et al.2021	Russia	Cohort study	SCC (lung)	517	7 years	Smoking cessation	Patients who quit smoking had a median overall survival time that was 21.6 months longer compared to those who continued smoking.
2.	Gemini et al 2019	UK	Cohort study	NSCLC (lung)	364	1 year	Smoking cessation	Smokers had poorer 12-month survival rates compared with never-smokers and ex-smokers.
3.	Koshiaris et al 2017	UK	Cohort study (Retrospective)	NS	191	NA	Smoking cessation	People who have lung cancer and stop smoking have a lower risk of death compared with people who continue to smoke.
4.	Ferketich et al 2013	US	Cohort study	SCC	1863	5 years	Smoking cessation	Those who were smoking around the time of diagnosis had poorer survival rates compared to those in their group who never smoked. Similarly, people suffering from stage IV diseases, current smokers had worse survival rates compared with former smokers having more than 12 months of quitting prior to the time of diagnosis.
5.	Mahdi Sheikh et al 2023	Russia	Cohort study	Renal cell Ca	212	8.2 years	Smoking cessation	Smoking cessation after the diagnosis of renal cell carcinoma significantly improves survival, reducing the risks of disease progression and cancer-specific mortality among smokers.
6.	Tse et al 2018	China	Cohort study	Lung (non specific)	3202	30 years	Smoking cessation	Among silicotics, quitting smoking for 10 years reduced lung cancer mortality by half, with the most pronounced benefits observed in patients with small opacities.

7.	Chen et al 2010	US	Cohort study	SCLC	163	Not specified	Smoking cessation	The research demonstrated the detrimental effects of smoking on the quality of life (QOL) of SCLC survivors and indicated that quitting smoking around the time of diagnosis may enhance overall QOL and alleviate symptoms.
8.	Chang et al 2014	Taiwan	Cohort study	Lung (non specific)	1677	10 years	Smoking cessation	Risks for former smokers who quit more than 5 years ago were similar to those for never smokers both for all-cause mortality and lung cancer.
9.	Kenfield et al 2011	US	Cohort study	prostrate	5366	8.1 years	Smoking cessation	Smoking at the time of prostate cancer diagnosis is associated with increased overall mortality, CVD mortality, as well as prostate cancer-specific mortality and recurrence. Prostate cancer specific mortality risks are comparable to those of never smokers for men who have been smoke-free for at least 10 years.
10.	Bian Z et al 2024	UK	Cohort	Mixed	37095	Not specified	Smoking cessation	In this multicohort study of survivors with cancer from the US, the UK, and China, a healthier lifestyle may improve prognosis after cancer diagnosis. Never smoking was strongly associated with a reduced risk of all-cause mortality.
11.	Heberg et al 2020	Denmark	Cohort	Mixed	7841	17 years	Smoking cessation	cancer survivors who quit smoking had a significantly lower risk of mortality compared to those who continued smoking. The findings indicate significant survival benefits from smoking cessation among female nurses who have survived cancer.
12.	Japuntich et al 2019	US	Cohort	Lung (non specific)	5575	7 years	Smoking cessation	In both lung cancer and colorectal cancer patients, current smokers at the time of diagnosis have higher mortality rates compared to never-smokers.

13.	Jang D et al 2024	Korea	Cohort study	Colorectal	37079	6.3 years	Smoking cessation	Smoking prior to a cancer diagnosis has a greater impact on the prognosis of colorectal cancer patients than smoking after diagnosis. Quitting smoking may enhance survival, particularly in patients with early-stage colorectal cancer
14.	Rades et al 2007	Germany	cohort	SCC	181	Not specified	Smoking cessation	Smoking during radiotherapy had a significant impact on local-regional control (LRC) and metastasis-free survival in patients with small cell lung carcinoma.
15.	Dobson Amato et al 2015	US	Cohort	Lung (non specific)	250	3.4 years	Smoking cessation	Quitting smoking remains the most effective way to mitigate or eliminate the harmful health effects of tobacco, especially survival among lung cancer patients.
16.	Tao et al 2013	China	Cohort	Bladder cancer	107	5.3 years	Smoking cessation	Continued smoking after a cancer diagnosis significantly increased the risk of death by 59% among male cancer patients
17.	Linhas et al 2018	Portugal	Cohort	NSLC Lung cancer	97	3.4 years	Smoking cessation	In a retrospective study of patients with advanced non-small cell lung cancer (NSCLC), smoking cessation before chemotherapy was associated with significantly improved overall survival in SCC patients, emphasizing the importance of quitting smoking during treatment to improve prognosis.
18.	Jamrozik et al 2011	Australia	Cohort	Lung (non specific)	23861	10 years	Smoking cessation	Smoking cessation significantly reduces the risk of death from smoking-related diseases, with greater benefits the longer the time since quitting.
19.	Frost et al 2011	UK	Cohort	Lung (non specific)	98912	Upto 34 years	Smoking cessation	The study highlights that smoking prevention and cessation are crucial for asbestos workers, as the interaction between asbestos exposure and smoking significantly increases lung cancer mortality risk.

20. Wang X et al 2023	US	Cohort	NSLC Lung cancer	5594	Not specified	Smoking cessation	The cohort study of patients with NSCLC was able to show history of quitting smoking early was associated with lower mortality following a lung cancer diagnosis; the association between smoking history and overall survival may have varied depending on clinical stage at diagnosis, possibly related to different treatment regimens and efficacy associated with smoking exposure following diagnosis.
21. Le PH et al 2024	Vietnam	Cohort	Mixed	Mixed	Not specified	Smoking cessation	The waterpipe tobacco smoking significantly increases the risk of all-cause mortality, underscoring the need for targeted control measures in Vietnam and similar regions.

Table S4: Characteristics of studies associated with Alcohol Moderation:

	Study ID.	Country	Study Design	Cancer Type	Number of Participants	Follow up Duration	Intervention	main outcomes
1.	Jung EJ et al 2012	South Korea	Cohort study	Mixed	16320	9.3 years	Alcohol moderation	Alcohol consumption may elevate the risk of overall mortality and death from specific cancers with significant correlations between the amount of alcohol consumed and the risk of overall mortality, as well as mortality from lung, stomach, and liver cancers.
2.	Minami Y et al 2019	Japan	Cohort study	Breast cancer	1420	8.6 years	Alcohol moderation	Light drinkers were found to have a decreased risk of breast cancer-specific death.
3.	Hermans KEPE et al 2021	Netherlands	Cohort study	Mixed	120852	20.3 years	Alcohol moderation	alcohol consumption is associated with increased Cancer of unknown Primary developing risk.
4.	Vrieling Aet al 2012	Germany	Cohort study	Breast cancer	2522	5 years	Alcohol moderation	consumption of alcohol before diagnosis is non-linearly associated with increased cancer specific mortality
5.	Tamokashi A et al 2017	Japan	Cohort study	Colorectal cancer	540	5 years	Alcohol moderation	Alcohol may increase the cancer-specific mortality risk

6.	Pelser C et al 2014	USA	Cohort study	Colorectal cancer	5727	5 years	Alcohol moderation	This current study showed light alcohol drinking was significantly associated with a reduced risk of all-cause mortality
7.	Bian Z et al 2024	UK	Cohort study	Mixed	37095	Not specified	Alcohol moderation	This study found that light alcohol drinking was significantly associated with a lower risk of all-cause mortality
8.	Farris MS et al 2018	Canada	Cohort study	Prostate Cancer	829	19 years	Alcohol moderation	Post-diagnosis alcohol consumption was associated with increased mortality, specifically for prostate cancer-specific mortality.
9.	Yang B et al 2017	USA	Cohort study	Colorectal cancer	2458	8.2 years	Alcohol moderation	There was no association seen in this present study between alcohol consumption and all-cause mortality among participants with non metastatic colorectal cancer.
10.	Walter V et al 2016	Germany	Cohort study	Colorectal cancer	3121	4.8 years	Alcohol moderation	Pre diagnostic alcohol consumption and heavy drinking were linked to poorer survival rates after a CRC diagnosis compared to light drinking. The protective benefits of light alcohol consumption may be limited to wine, and these associations could vary based on age and the presence of diabetes mellitus
11.	Koyama S et al 2024	Japan	Cohort study	Oral & Pharyngeal	2626	10 years	Alcohol moderation	An increased risk of mortality for heavy drinkers consuming more than 46 g/day of ethanol

								compared to non-consumers was observed in both men and women
12.	Benyon RA et al 2018	UK	Cohort study	Head & Neck	1393	3.5 years	Alcohol moderation	They found no evidence that individuals who consumed hazardous to harmful amounts of alcohol at the time of diagnosis had a higher risk of mortality compared to non-drinkers.
13.	Ramadas K et al 2010	India	Cohort study	Mixed	167343	6.5 years	Alcohol moderation	Smoking and alcohol uses were associated with increased risk of all-cause, all cancer and tobacco-related cancer mortality
14.	Denissoff A et al 2022	Finland	Cohort study	Head & Neck	1033	5 years	Alcohol moderation	In this study alcohol consumption was found to increase risk of mortality
15.	Din N et al 2016	USA	Cohort study	Breast Cancer	939	11 years	Alcohol moderation	They found an association between alcohol consumption and breast cancer-specific mortality among current smokers
16.	Yi SW et al 2010	South Korea	Cohort study	Esophageal Ca	6291	20.8 years	Alcohol moderation	The study found that alcohol consumption significantly increases mortality risk from esophageal cancers in men, highlighting the importance of moderation in alcohol intake.
17.	Jayasekara H et al 2018	Australia	Cohort study	Colorectal Ca	724	9 years	Alcohol moderation	The study noted a positive correlation between alcohol consumption and increased mortality from colon and bile duct cancers, highlighting the necessity for moderation.

18.	Jankhotkaew J et al 2020	Thailand	Cohort study	Mixed	59312	30 years	Alcohol moderation	Regular drinking of alcohol increased the risk for all-cause and cancer mortality. Interventions should be given to reduce the number of regular drinkers in order to save one's life.
19.	McCain RS et al 2020	North Ireland	Cohort study	Esophageal Ca	130	2.5 years	Alcohol moderation	While in some biomarker-selected subgroups, ever-alcohol consumption was associated with worsened survival compared with never drinkers.
20.	Zeinomar N et al 2023	US	Cohort study	Breast Ca	1926	6.7 years	Alcohol moderation	No statistically significant relation of alcohol consumption was found with all-cause mortality (>3 drinks per week vs non-drinkers: HR, 1.05; 95% CI, 0.73-1.51) and breast cancer-specific mortality (>3 drinks per week vs non-drinkers: HR, 1.06; 95% CI, 0.67-1.67).
21.	Mayne ST et al 2009	US	Cohort study	Oral & Pharyngeal Ca	264	4.2 years	Alcohol moderation	Continued alcohol consumption after a diagnosis of head and neck cancer has an adverse impact on survival. Steps for cessation should be part of the survivorship care for these patients.

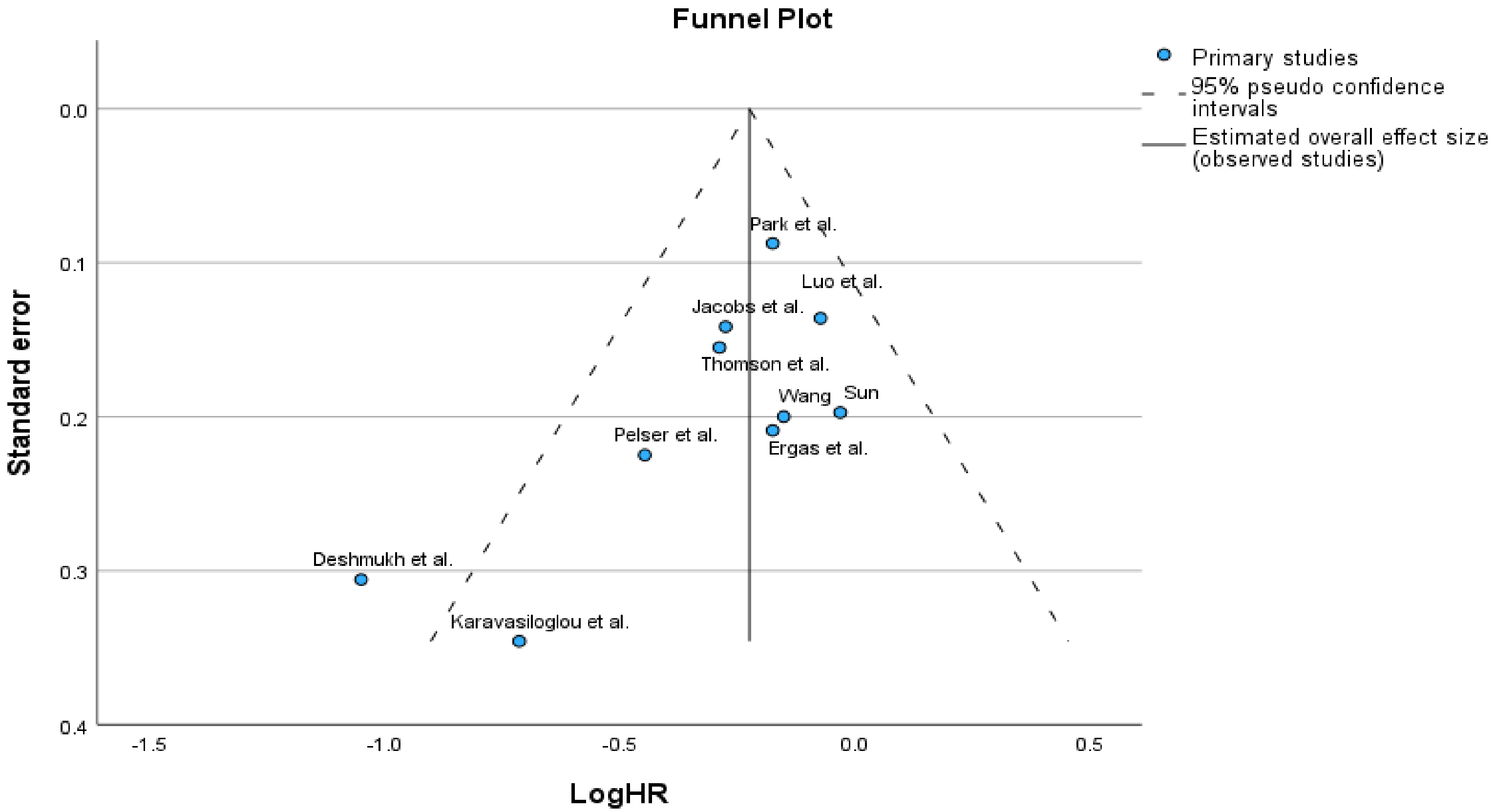

Figure S1: Funnel plots to assess publication bias in studies assessing HEI diet impact on cancer survival

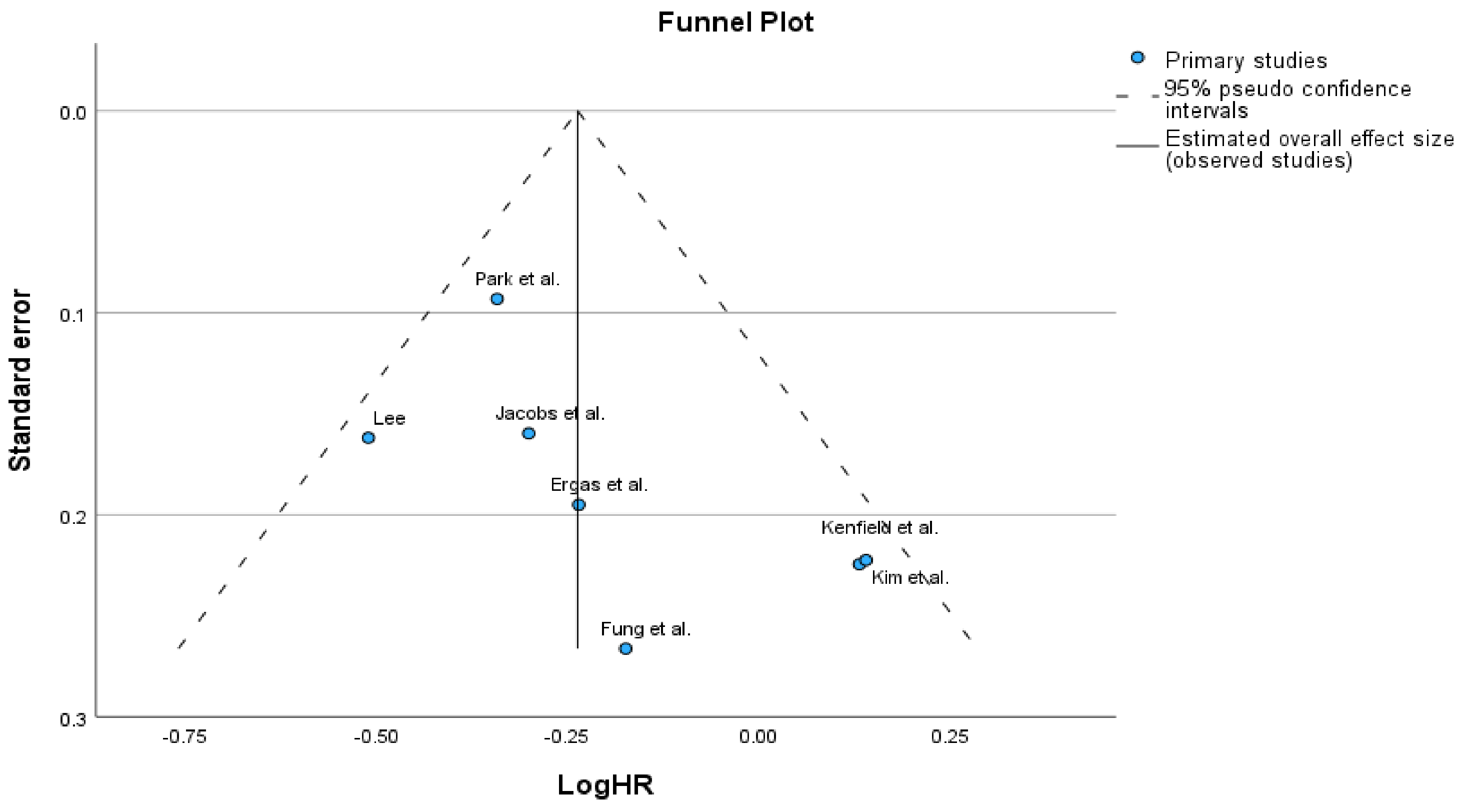

Figure S2: Funnel plots to assess publication bias in studies assessing aMED diet impact on cancer survival.

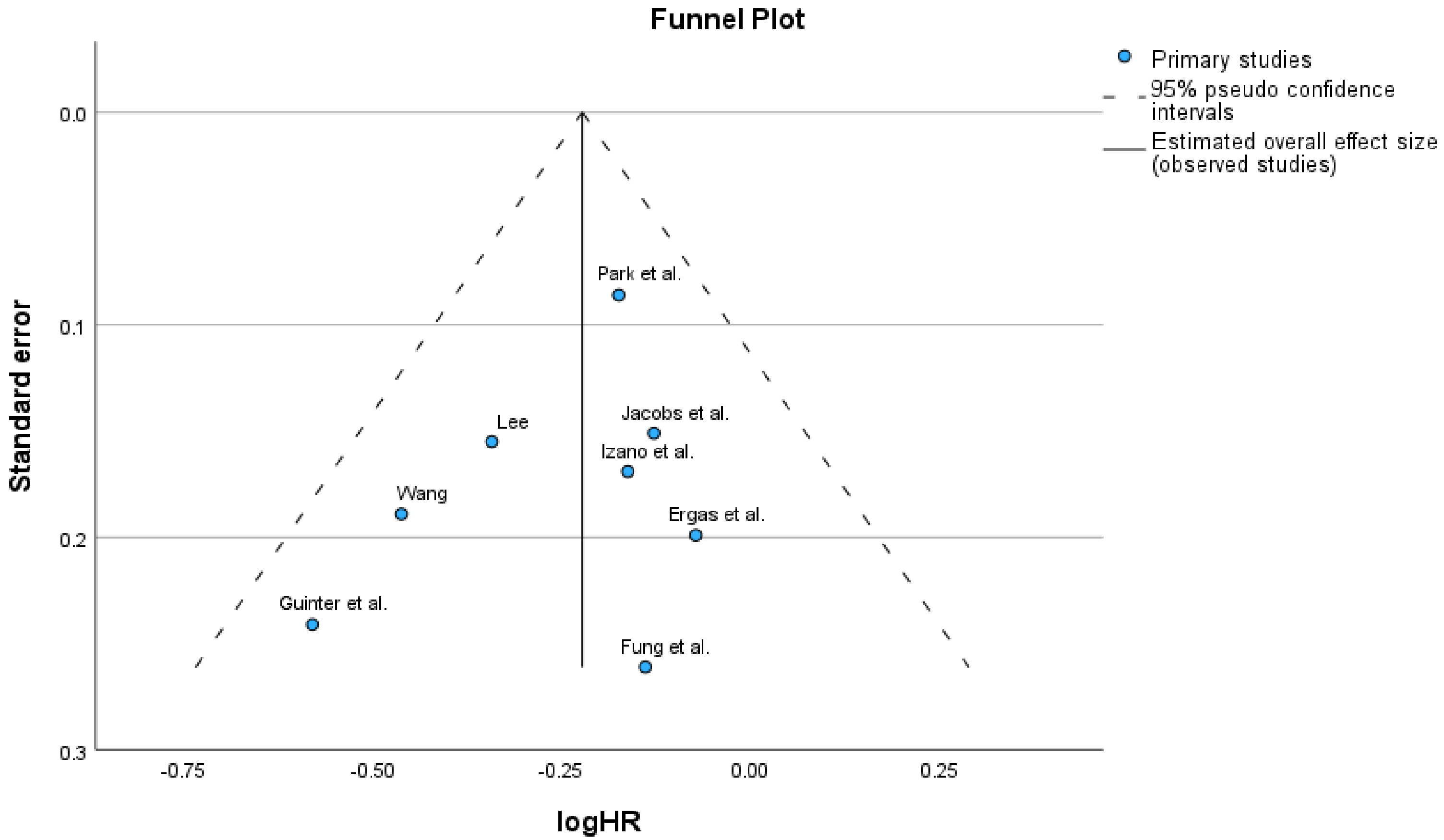

Figure S3: Funnel plots to assess publication bias in studies assessing DASH diet impact on cancer survival.

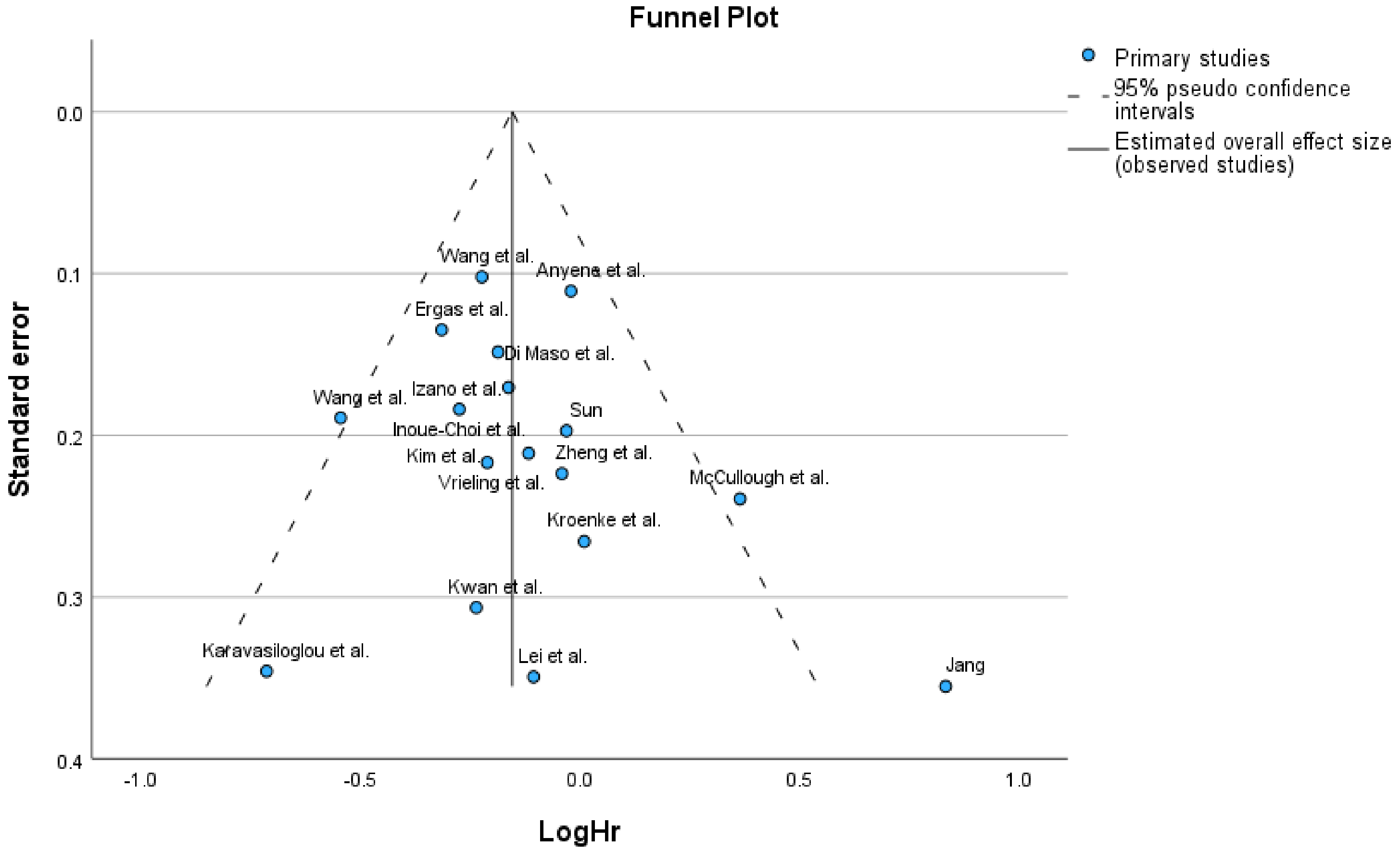

Figure S4: Funnel plots to assess publication bias in studies assessing diet impact on breast cancer survival.

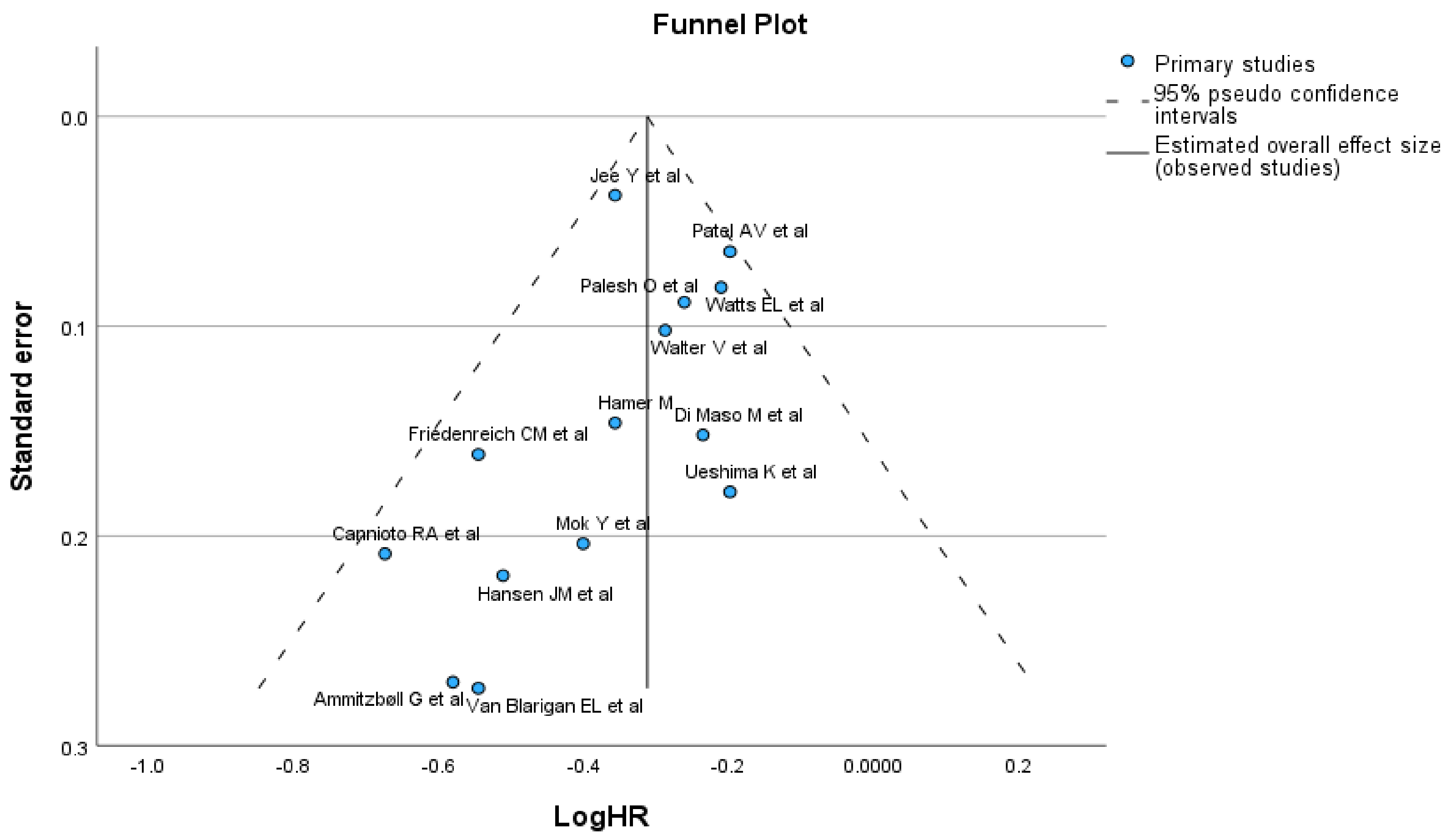

Figure S5: Funnel plot to assess publication bias in studies assessing physical activity impact on cancer outcomes.

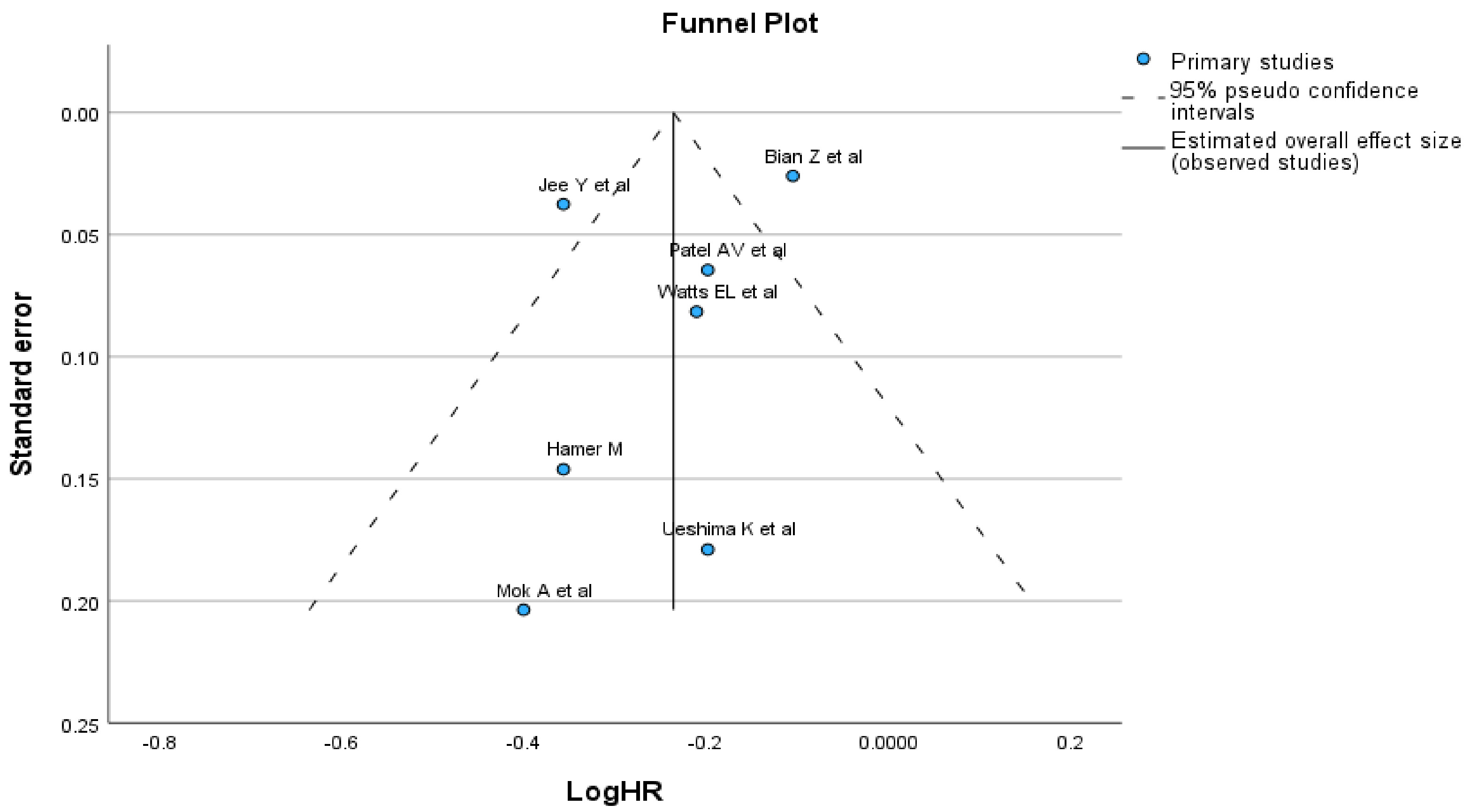

Figure S6: Funnel plot to assess publication bias in studies assessing physical activity impact on all cancer survival outcomes only.

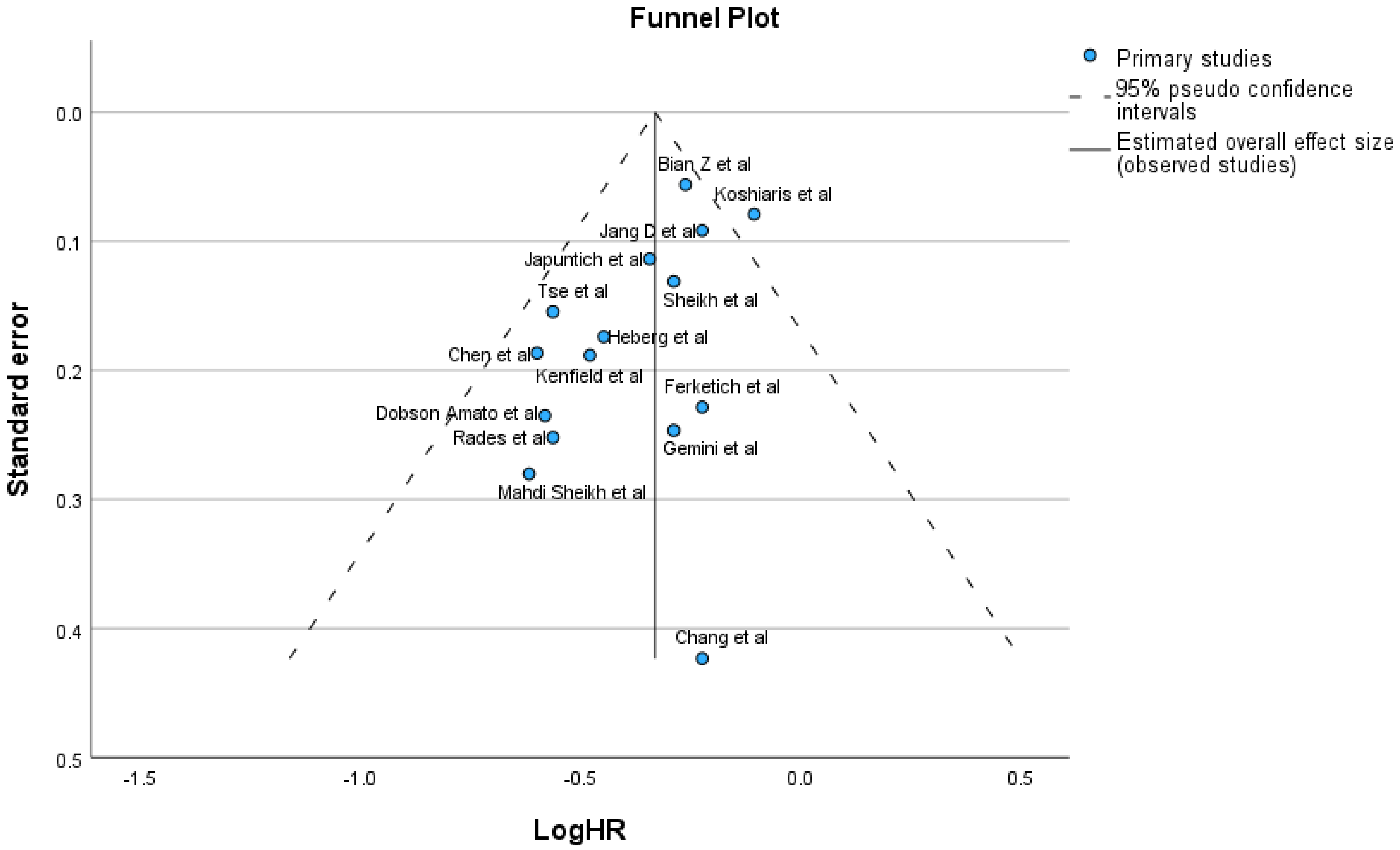

Figure S7: Funnel plot to assess publication bias in studies assessing smoking cessation impact on all cancer survival.

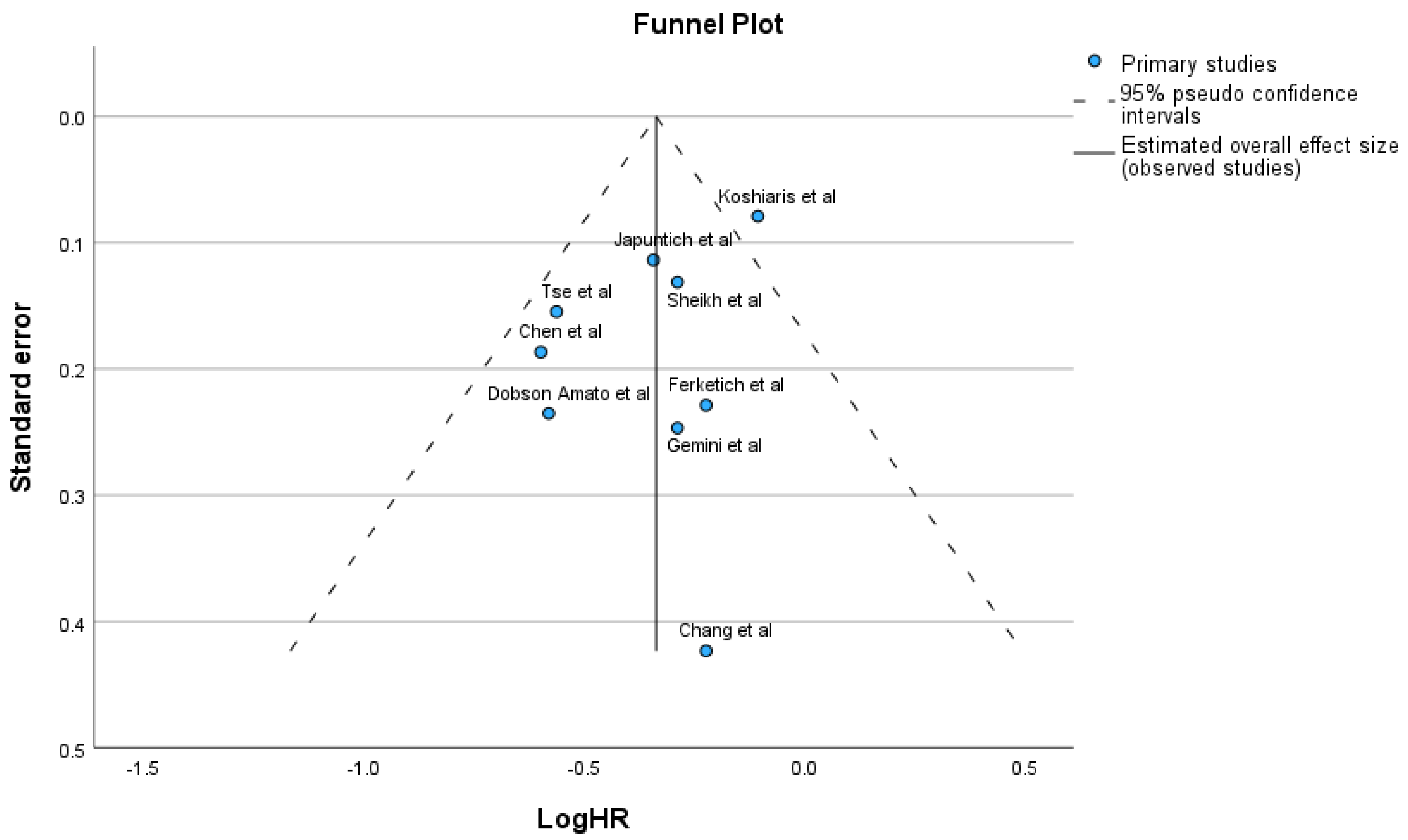

Figure S8: Funnel plot to assess publication bias in studies assessing smoking cessation impact on lung cancer survival.

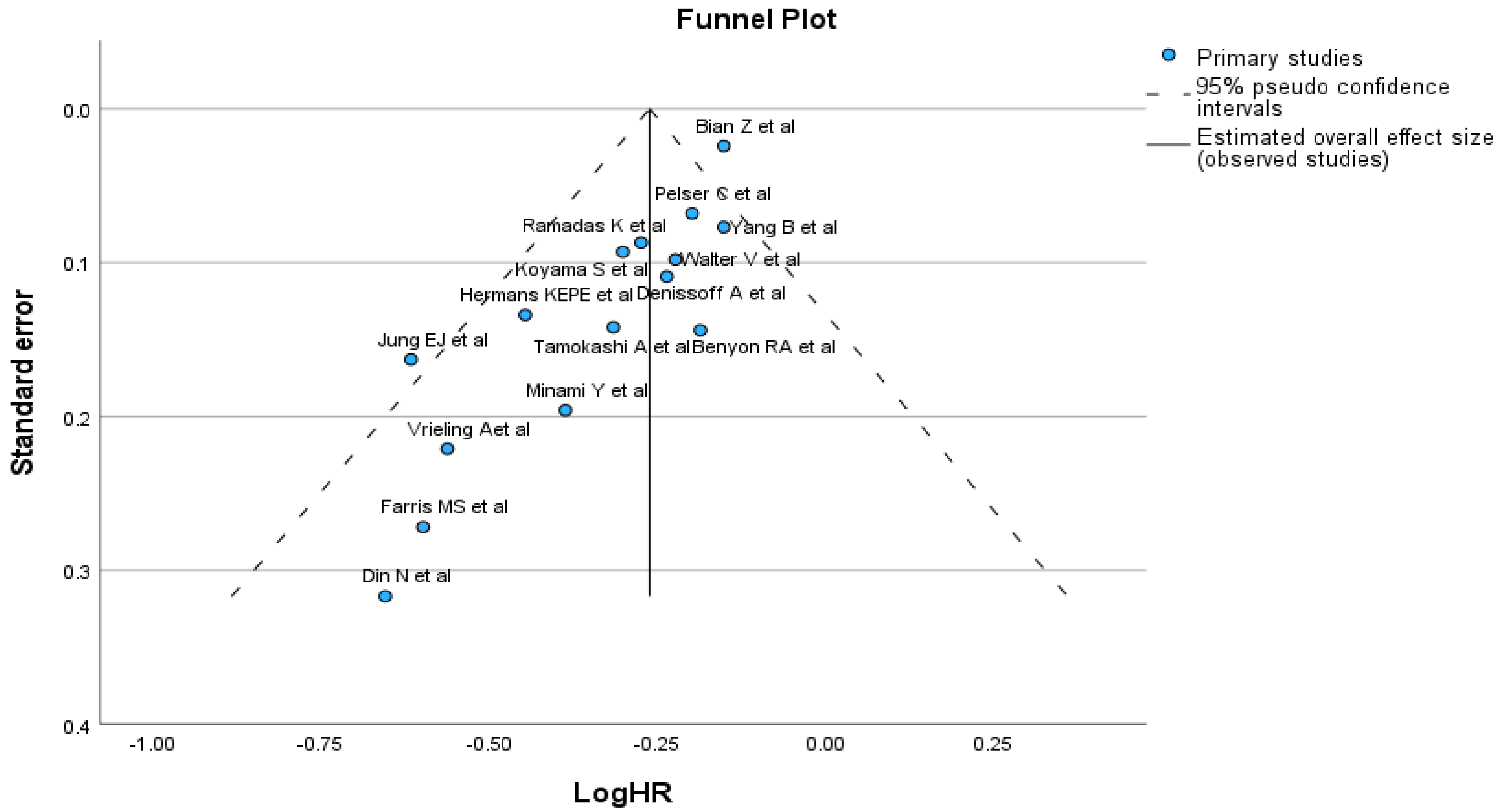

Figure S9: Funnel plot to assess publication bias in studies assessing alcohol impact on all cancer survival.

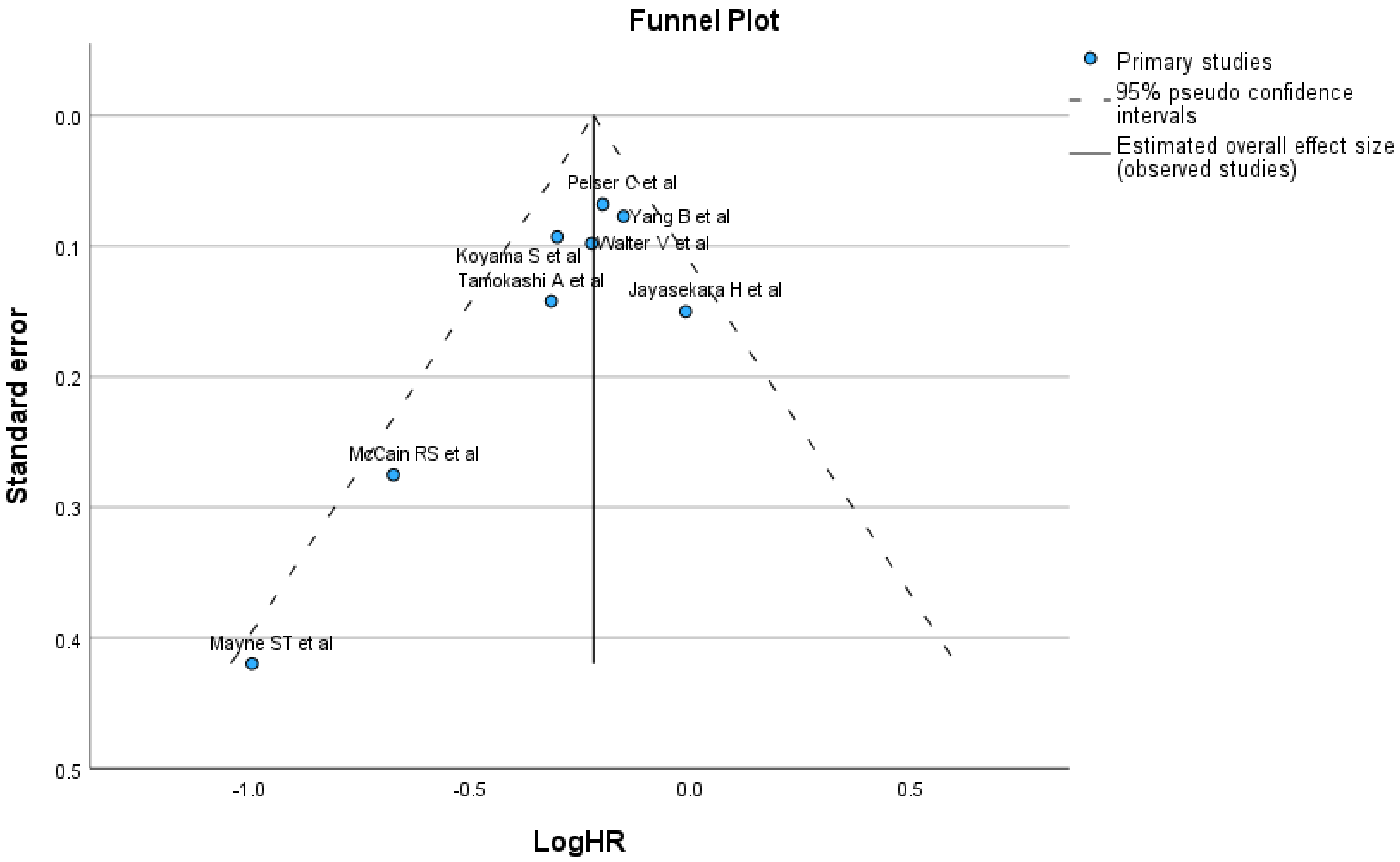

Figure S10: Funnel plot to assess publication bias in studies assessing alcohol impact on Cancers of gastrointestinal tract survivals

SEARCH QUERIES FOR DIFFERENT DATABASES

PUBMED SEARCH QUERY

("cancer mortality"[Title/Abstract] OR "cancer survival"[Title/Abstract] OR "neoplasms/mortality"[MeSH Terms] OR "long-term survival"[Title/Abstract] OR "cancer recurrence"[Title/Abstract]) AND ("lifestyle modification"[Title/Abstract] OR "life style"[MeSH Terms] OR "diet"[Title/Abstract] OR "Diet Therapy"[MeSH Terms] OR "dietary intervention"[Title/Abstract] OR "physical activity"[Title/Abstract] OR "Motor Activity"[MeSH Terms] OR "exercise"[Title/Abstract] OR "Smoking Cessation"[Title/Abstract] OR "Smoking Cessation"[MeSH Terms] OR "alcohol consumption"[Title/Abstract] OR "Alcohol Drinking"[MeSH Terms] OR "alcohol moderation"[Title/Abstract])

SCOPUS SEARCH QUERY

TITLE-ABS-KEY (("cancer mortality" OR "cancer survival" OR "cancer death" OR "oncological outcome" OR "long-term survival" OR "cancer recurrence") AND ("lifestyle modification" OR "life style" OR diet OR "diet therapy" OR "dietary intervention" OR nutrition OR "nutritional intervention" OR "physical activity" OR "motor activity" OR exercise OR "smoking cessation" OR "quit smoking" OR "tobacco cessation" OR "alcohol consumption" OR "alcohol drinking" OR "alcohol moderation"))

COCHRANE SEARCH QUERY

("cancer mortality" OR "cancer survival" OR "cancer death" OR "oncological outcome" OR "long-term survival" OR "cancer recurrence") AND ("lifestyle modification" OR "life style" OR diet OR "diet therapy" OR "dietary intervention" OR nutrition OR "nutritional intervention" OR "physical activity" OR "motor activity" OR exercise OR "smoking cessation" OR "quit smoking"

OR "tobacco cessation" OR "alcohol consumption" OR "alcohol drinking" OR "alcohol moderation")
